# Cancer Neoepitopes for Immunotherapy: Discordance Between Tumor-Infiltrating T Cell Reactivity and Tumor MHC Peptidome Display

**DOI:** 10.3389/fimmu.2019.02766

**Published:** 2019-12-11

**Authors:** Stina L. Wickström, Tanja Lövgren, Michael Volkmar, Bruce Reinhold, Jonathan S. Duke-Cohan, Laura Hartmann, Janina Rebmann, Anja Mueller, Jeroen Melief, Roeltje Maas, Maarten Ligtenberg, Johan Hansson, Rienk Offringa, Barbara Seliger, Isabel Poschke, Ellis L. Reinherz, Rolf Kiessling

**Affiliations:** ^1^Department of Oncology and Pathology, Karolinska Institutet, Stockholm, Sweden; ^2^Department of Immunology, Genetics and Pathology, Uppsala University, Uppsala, Sweden; ^3^Division of Molecular Oncology of Gastrointestinal Tumors, German Cancer Research Center (DKFZ), Heidelberg, Germany; ^4^Laboratory of Immunobiology, Dana-Farber Cancer Institute, Boston, MA, United States; ^5^Department of Medical Oncology, Dana-Farber Cancer Institute and Department of Medicine, Harvard Medical School, Boston, MA, United States; ^6^Institute of Medical Immunology, Martin Luther University Halle-Wittenberg, Halle, Germany; ^7^DKTK Immune Monitoring Unit, German Cancer Research Center (DKFZ) and National Center for Tumor Diseases (NCT), Heidelberg, Germany

**Keywords:** TIL, tumor, Immune peptidome, neoepitopes, mass spectrometry, immunotherapy

## Abstract

Tumor-infiltrating lymphocytes (TIL) are considered enriched for T cells recognizing shared tumor antigens or mutation-derived neoepitopes. We performed exome sequencing and HLA-A^*^02:01 epitope prediction from tumor cell lines from two HLA-A2-positive melanoma patients whose TIL displayed strong tumor reactivity. The potential neoepitopes were screened for recognition using autologous TIL by immunological assays and presentation on tumor major histocompatibility complex class I (MHC-I) molecules by Poisson detection mass spectrometry (MS). TIL from the patients recognized 5/181 and 3/49 of the predicted neoepitopes, respectively. MS screening detected 3/181 neoepitopes on tumor MHC-I from the first patient but only one was also among those recognized by TIL. Consequently, TIL enriched for neoepitope specificity failed to recognize tumor cells, despite being activated by peptides. For the second patient, only after IFN-γ treatment of the tumor cells was one of 49 predicted neoepitopes detected by MS, and this coincided with recognition by TIL sorted for the same specificity. Importantly, specific T cells could be expanded from patient and donor peripheral blood mononuclear cells (PBMC) for all neoepitopes recognized by TIL and/or detected on tumor MHC-I. In summary, stimulating the appropriate inflammatory environment within tumors may promote neoepitope MHC presentation while expanding T cells in blood may circumvent lack of specific TIL. The discordance in detection between physical and functional methods revealed here can be rationalized and used to improve neoantigen-targeted T cell immunotherapy.

## Introduction

Tumor-infiltrating lymphocytes (TIL) in patients with metastatic malignant melanoma are thought to be enriched for T cells that can recognize antigens expressed by the patient's tumor. In line with this, therapy with autologous TIL, expanded to large numbers *ex vivo* and reinfused to melanoma patients, can induce long-lasting clinical responses in a large proportion (40–70%) of patients (1). Different categories of tumor-associated antigens (TAA) are recognized by TIL, and initial efforts focused on broadly expressed TAA shared between patients. Such TAA include both differentiation antigens that are found in the normal melanocytic counterparts and aberrantly expressed antigens such as cancer-testis antigens that are normally expressed only in immune privileged sites. Therapeutic approaches with T cells transduced with T cell receptors (TCR) recognizing these types of shared TAA, exemplified by NY-ESO-1, MART-1, gp100, and MAGE-A3, have resulted in clinical regressions of metastatic lesions in a limited number of treated patients, sometimes with severe side effects caused by cross-reactivity to normal tissues (2, 3).

Recently, the focus of the research field has shifted toward tumor-specific antigens associated with somatic mutations (neoantigens/neoepitopes), which are in the majority of cases unique for each patient. This development has been spurred by advancements in next-generation sequencing (NGS) techniques that have made it possible to almost routinely identify all tumor-associated mutations, including both shared mutations in driver genes (e.g., Ras, p53) and patient-unique passenger mutations. Passenger mutations are not part of oncogenesis, but tend to accumulate during tumor progression especially in tumors caused by UV or carcinogen exposure, typically exemplified by melanomas, and lung cancers.

Neoepitopes resulting from mutations are attractive cancer immunotherapy targets. The mutation is not present during the selection in the thymus and thus exempt from central tolerance. Thus, neoepitopes are seen as “foreign” non-self. In addition, the mutations are truly tumor-specific and there is less risk for ON-target, OFF-tumor side effects although cross-reactivities to epitopes in other proteins can probably occur. Several lines of evidence have indicated that neoepitope frequency can be decisive in determining the capacity of patient's T cells to reject their tumors. Thus, an association between mutational load and clinical outcome in patients treated with antibodies blocking the checkpoint molecules CTLA4 and PD-1 has been described (4, 5). In addition, a connection between clinical efficacy of TIL adoptive cell therapy (ACT) and the presence of T cells specific for tumor-derived mutations in the infused TIL has been suggested (6, 7). Furthermore, ACT performed with TIL enriched for neoepitope-specific T cells has resulted in successful clinical outcomes (8, 9).

In this study, we used two peptide libraries containing *in silico*-predicted T cell neoepitopes derived from whole exome sequencing data of early passage tumor cell lines from two HLA-A2^*^02:01-positive melanoma patients. The predicted neoepitopes were screened for their ability to activate autologous TIL in functional assays and for their presence on MHC-I by mass spectrometry (MS). This combined approach revealed a significant discordance between the immunologic and physical detection methods, with neoepitopes recognized by autologous TIL not being detected by MS, and vice versa. Here, this discrepancy is examined. Peptide recovery and detection sensitivity for MS are characterized in addition to TIL functional assays including assessment of specificity, avidity, and activation capacity, as well as neoepitope immunogenicity. Our results highlight the difficulties to be faced when aiming to target tumors with neoepitope-specific, T cell-based immunotherapy and suggest strategies on how to improve such therapy.

## Materials and Methods

### Patients

Patient ANRU is a male born in 1975 who was operated in November 2014 for stage III axillary lymph node metastatic melanoma from which a tumor line and TIL cells were isolated. He had a relapse with CNS metastases in 2015, for which he was operated and received local radiotherapy. Since then, he is tumor free and has received no systemic therapy. Patient KADA is a male born in 1938 who was operated in 2011 for stage III axillary lymph node metastasis from which a tumor line and TIL cells were isolated. He has had no systemic treatment and has since then remained recurrence free. The protocol for patient participation was approved by the local Ethics Committee (Dno. 2011/143-32/1 and 2015/1862-32) and the Institutional Review Board. Both patients signed a written informed consent in accordance with the Declaration of Helsinki.

### Cells and Tissues

Original SK-OV-3, HLA-A2^*^02:01-transfected SK-OV-3 and T2 cell lines were grown in RPMI supplemented with FCS (10%), penicillin (100 U/ml; LifeTechnologies), and streptomycin (100 μg/ml; LifeTechnologies). PBMC were prepared from healthy blood donor buffy coats by Ficoll-Hypaque (GE Healthcare) density-gradient centrifugation, according to the manufacturer's instructions.

Monocytes and CD8+ T cells were isolated using CD14+ or CD8+ magnetic-bead-based isolation (Miltenyi Biotec), respectively, according to the manufacturer's instructions. Monocytes were matured into dendritic cells (DC) using a two-step protocol as described previously (10).

Resected tumors from two HLA-A2^*^02:01-positive melanoma patients (acronym ANRU and KADA) were used for generation of tumor cell lines and expansion of TIL. Tumor cell lines were established by mechanical dissociation of tumor tissue by cutting and grinding through a 70-μm cell strainer (Corning). Tumor cells were cultured in RPMI (LifeTechnologies) supplemented with FCS (20%), penicillin (100 U/ml; LifeTechnologies), and streptomycin (100 μg/ml; LifeTechnologies). Tumor cells were monitored frequently for growth and medium was changed/cultures were expanded when necessary. Where indicated, tumor cells were first cultured for 24 h using standard culture conditions and were thereafter exposed to IFN-γ (25 ng/ml; R&D Systems) for 72 h.

TIL were expanded as described by Poschke et al. (11), by first stimulating expansion from tumor fragments with IL-2 alone and thereafter performing a rapid expansion protocol (REP) by stimulation with IL-2 and anti-CD3 antibodies in the presence of irradiated PBMC as feeder cells. All TIL and T cells, including co-cultures, were cultured in CellGro® plus human AB-serum (2%; The Blood Bank, Karolinska University Hospital).

### Exome Sequencing Data Analysis

See Supplementary Materials and Methods.

### Expression Analysis of Mutated Genes and Alleles

To coarsely estimate expression of mutated genes in the absence of whole transcriptome data for the tumor cell lines, publicly available RNA-seq profiles of seven melanoma cell lines [GSE46817 (12, 13)] and averaged reads per kilo base per million mapped reads (RPKM) expression values were collected and analyzed. Genes with mean RPKM >1 and a low standard deviation were considered expressed.

To verify transcription of selected genes and mutated/wild-type alleles, total RNA from ANRU and KADA tumor cell lines was isolated using the RNeasy Mini kit (Qiagen) and quality and quantity were measured on an Agilent 2100 Bioanalyzer (Agilent Technologies, Santa Clara, CA, USA). To confirm expression of the mutated alleles, isolated RNA was treated with DNase (Thermo Fisher Scientific, Waltham, MA USA) and converted to cDNA using SuperScript^TM^ III reverse transcriptase (RT; Thermo Fisher Scientific) and oligo(dT)_15_ primers. For each sample, a control without the reverse transcriptase was included to detect possible contaminating genomic DNA. The selected genes were amplified using MyTaq DNA polymerase (Bioline, London, UK) and PCR primers listed in Supplementary Table 4. The PCR products were sent for Sanger sequencing (Eurofins MWG GmbH, Ebersberg, Germany) and allelic expression was assessed from the resulting electropherograms.

To quantify total expression of selected genes and the common melanoma tumor-associated antigens MART-1 and gp100, relative to housekeeping genes GAPDH and β-actin [primers from Radonić et al. (14)], quantitative PCR (qPCR) was performed using the LightCycler® 480 SYBR Green I Master and a Roche LightCycler® 480. Amplification efficiency was verified with serial dilutions of template cDNA. All samples were amplified in triplicate and resulting Cp values were averaged.

### Peptides and Peptide Libraries

Neoepitopes with a predicted affinity of <50 nM and mean RPKM of at least 1 were ordered as crude micro-scale peptide libraries (JPT Peptide Technologies). The KADA library contained 181 peptides and the ANRU library contained 49 peptides. Neoepitope peptides found to activate TIL or that were found on tumor MHC-I were thereafter ordered in larger scale and higher purity, as were corresponding wild-type peptides.

### Analysis of MHC-I Presentation Machinery

The analysis of MHC presentation machinery was performed as previously described (15) by Western blot staining for peptide processing components (TAP1 and 2, Tapasin, MHC-I heavy chain, LMP2 and 10, and β2m) in untreated and IFN-γ-treated tumor cells, confirmed also by quantitative PCR.

### Isolation of Peptide-HLA Class I Complexes From Melanoma Cell Lines

See Supplementary Materials and Methods.

### Immune Peptidome Analysis by MS

All Poisson detection MS methodology and liquid chromatography (LC) data independent acquisition (DIA) MS methodologies have been described in detail previously (16–18). In brief, mass spectra were collected on a quadrupole-TOF (Sciex 6600+) instrument in a DIA format. The *m*/*z* region 400–680 was split into 11 minimally overlapping windows of variable width designed to transmit equal ion fluxes with MHC-I immune peptidomes. MS data were collected in a series of a single full-range MS spectrum followed with 11 MS/MS spectra for each transmitted window. The MS/MS spectra were compared with reference patterns obtained from synthetic peptides using an algorithm based on the theory of sampling a Poisson process (18). High LC-MS sensitivity was promoted using electrospray ionization with 20 μm ID alkane modified polystyrene-divinylbenzene monolithic columns [fabricated in-house (19)] at flow rates of roughly 10 nl/min. Elution positions of the synthetic peptides relative to shared endogenous immune peptides using the same column configuration were also determined, and this provides a restrictive map for the elution positions of the neoepitope candidates in the tumor DIA MS data (Supplementary Figure 5).

### TIL Functional Assessment

Recognition of tumor cells or neoepitope-pulsed antigen-presenting cells (APC; SK-OV-3) by TIL was assessed in co-cultures using a TIL:tumor cell/APC ratio of 5:1 in 96-well U-bottom plates. Recognition of neoepitope peptides by TIL was first tested using pools of 5 (ANRU) or 10 (KADA) peptides. For pools that activated TIL, each peptide was tested individually. Known shared TAA epitopes (MART-1: ELAGIGILTV, MAGE-A4: GVYDGREHTV, MAGE-A10: GLYDGMEHL, gp100: IMDQVPFSV, tyrosinase: YMDGTMSQV, NY-ESO-1: SLLMWITQV) or viral epitopes (CMV pp65 antigen: NLVPMVATV, HCV NS3 antigen: KLVALGINAV, HIV p17 antigen: SLYNTVATL, Influenza M1 antigen: GILGFVFTL) were included as controls.

Peptide recognition was analyzed by pulsing of HLA-A2-transfected SK-OV-3 cells with 10 μg/ml total peptide concentration (resulting in 2 or 1 μg/ml of each peptide in the 5- and 10-peptide pools, respectively) in PBS for 1 h at 37°C before washing and co-culture. Original untransfected SK-OV-3 were included as negative controls. For KADA, due to too high background stimulation by SK-OV-3-A2+ cells, peptide pools (10 μg/ml) were added directly to KADA TIL, which then served as APC themselves. Thereafter, single peptides from pools that activated KADA TIL were analyzed using HLA-A2-transfected SK-OV-3 as APC, as described. Where indicated, MHC-I was blocked by pre-incubating tumor cells or APC for 30 min at 37°C with 20 μg/ml anti-HLA-ABC antibody (clone W6/32, BioLegend) or anti-HLA-A2 antibody (clone BB7.2, AbD Serotec) before addition of TIL. CD3/CD28 Dynabeads (LifeTechnologies) were used as positive control according to the manufacturer's instructions.

Readouts for TIL activation were degranulation measured as surface expression of CD107a and cytokine production as measured by intracellular or secreted IFN-γ by FACS or ELISA, respectively. Experiments aiming to determine T cell functional avidity were performed by titrating peptides in eight steps of 10-fold dilutions from 100 μg/ml directly on TIL.

In experiments where CD107a and dextramer staining was to be performed, co-cultures were incubated for 5 h before being harvested for staining. In experiments with CD107a staining but without dextramer staining, GolgiPlug^TM^ and GolgiStop^TM^ (BD Bioscience) were added after 2 h co-culture, and cells were harvested after an additional 4 h co-culture.

In experiments where IFN-γ ELISA analysis of supernatants was to be performed, co-cultures were incubated for 24 h.

### Flow Cytometry

All antibodies and other flow cytometry reagents were used according to the manufacturer's instructions, unless otherwise stated. All had been titrated for optimal signal-to-noise ratio and staining was, unless specified differently, performed in PBS supplemented with 0.1% albumin. Data for all flow cytometry were acquired on a NovoCyte (ACEA Biosciences) or a BD LSR II (BD Biosciences) and analyzed using FlowJo Software (TreeStar) as geometric MFI or percent positive cells compared to the parent population.

Staining was performed for T cells specific for known shared tumor-associated antigen T cell epitopes (MART-1, NY-ESO-1, MAGE-A3, Tyrosinase, gp100, and MAGE-A1; Melanoma Dextramer® Collection 1 kit, Immudex) or for neoepitope-specific T cells (custom-ordered PE-labeled neoepitope/HLA-A2^*^02:01 dextramers, Immudex). Cell surface expression was analyzed for CD8 (clone SK1, APC-Cy7, BioLegend), CD3 (clone UCHT1, PE-Cy7, BioLegend), MHC-I (clone W6/32, APC, BioLegend), and HLA-A2 (clone BB7.2, PE, BioLegend). All staining protocols included a dead cell marker (staining in PBS only; LIVE/DEAD® fixable Aqua Dead cell stain, InVitrogen).

Detection of activated T cells was performed by staining with CD107a antibody (clone H4A3, FITC, BioLegend), which was added to stimulated TIL/T cell cultures at experiment setup (20).

When dextramer staining was performed, dextramers were always added first, then CD8 staining and last dead cell labeling. In experiments where intracellular staining was performed, cells were stained for dead cells, then CD3 and CD8, before fixation and permeabilization using CytoPerm/CytoFix^TM^ (BD Biosciences) and intracellular staining for IFN-γ (clone 4S.B3, PE, Biolegend).

### HLA-A2 Stabilization Assay

HLA-A2 stabilization assays were performed using T2 cells that were harvested, washed, resuspended in serum-free RPMI and thereafter seeded at 200,000 cells/well in 96 U-bottom plates (TPP®). Peptides were added in serial dilution of 1.5–100 μg/ml and incubated overnight in room temperature and then an additional 2.5 h at 37°C. The cells were then harvested and stained for HLA-A2 as described above. HLA-A2 stabilization data were normalized according to the formula (gMFI (peptide) – gMFI (no peptide))/gMFI (no peptide).

### Generation of Neoepitope-Specific TIL Lines or Stimulation of Neoepitope-Specific Cells From PBMC

TIL were sorted for neoepitope specific T cells by labeling with custom PE-labeled neoepitope dextramers, as described. The dextramer-stained cells were enriched by MACS by binding to anti-PE microbeads (Miltenyi Biotec), following the manufacturer's instructions. Both enriched and depleted populations were immediately subjected to a REP to expand TIL. As control, unsorted TIL were subjected to REP in parallel.

To expand neoepitope-specific cells from patient or HLA-A2+ donor blood, DC were loaded with neoepitope peptides as described for SK-OV-3 cells above. The DC were co-cultured with autologous CD8+ T cells in a 1:5 ratio for 14 days in CellGro® supplemented with 20 IU/ml IL-2 (Proleukine, Novartis).

### Immunoassays

ELISA for IFN-γ (MabTech) was performed according to the manufacturer's instructions. Standard curves were plotted as four-parameter sigmoidal curves and unknowns were calculated and plotted using GraphPad Prism (GraphPad).

## Results

### Recognition of Tumor Cells and Common Melanoma TAA by TIL

**Figure 1:**
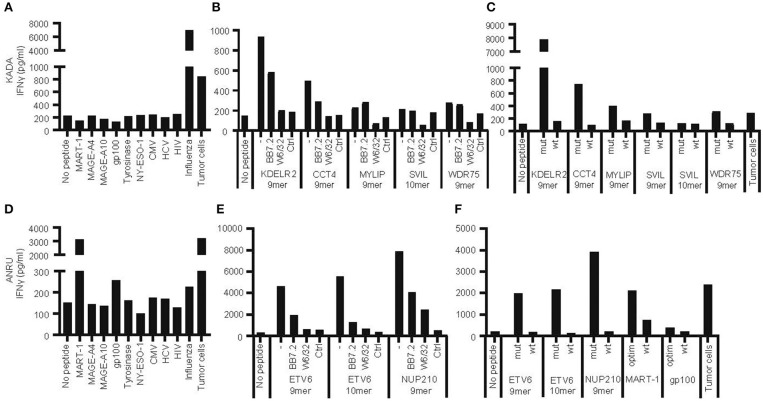
Recognition of autologous tumor cells or peptides from shared tumor-associated antigens, from mutated genes, or from viruses by tumor-infiltrating lymphocytes. Tumor-infiltrating lymphocytes (TIL) and primary tumor cell lines were expanded from tumors of patients KADA and ANRU. The ability of TIL to recognize corresponding tumor cells or HLA-A2-transfected SK-OV-3 target cells pulsed with peptides from common tumor-associated antigens or viruses (**A**, KADA; **D**, ANRU), or HLA-A2-transfected SK-OV-3 cells pulsed with neoepitope peptides derived from mutated genes in tumor cells, in the absence or presence of anti-HLA-A2 (BB7.4) or -MHC-I (W6/32) blocking monoclonal antibodies (**B**, KADA; **E**, ANRU; only recognized peptides shown), or HLA-A2-transfected SK-OV-3 pulsed with neoepitope peptides compared to corresponding wild-type peptides (**C**, KADA; **F**, ANRU), and respond with IFN-γ secretion was assessed after 24 h by ELISA. Unpulsed HLA-A2-transfected SK-OV-3 target cells were used as a no peptide control and pulsed original SK-OV-3 cells (Ctrl) were used as a no HLA-A2 control as indicated.

Tumor cell lines and corresponding TIL were generated from two HLA-A^*^02:01-positive melanoma patients (KADA and ANRU). Both TIL recognized autologous tumor cells and responded with degranulation and production of IFN-γ, which was measured by FACS as increased cell surface CD107a and intracellular IFN-γ (Supplementary Figure 1A) or by ELISA as secreted IFN-γ in supernatants [Figure 1A (KADA) and Figure 1D (ANRU)]. The activation was partly decreased by blocking HLA-A2 (BB7.2 mAb) and more pronouncedly by total blocking of MHC-I (W6/32 mAb) on tumor cells (Supplementary Figure 1A).

Next, we evaluated whether activation of TIL was due to recognition of some of the TAA commonly expressed by melanoma [Figure 1A (KADA) and Figure 1D (ANRU)] by co-culture of TIL and peptide-pulsed HLA-A2-transfected SK-OV-3 target cells. KADA TIL were only activated by a control peptide derived from the Influenza M1 matrix protein, while ANRU TIL were activated by peptides from both MART-1 and, although weaker, gp100. This specificity of ANRU TIL for MART-1 and weakly for gp100 was confirmed by FACS analysis using a panel of dextramers for common melanoma antigens (MART-1, NY-ESO-1, MAGE-A3, Tyrosinase, gp100, and MAGE-A1; Supplementary Figure 1B). Of note, both these peptides were tested in their modified form, optimized for better HLA-A2 binding, while the native peptides have a much lower affinity for HLA-A2 (**Table 2**) and also a decreased ability to activate T cells (Figure 1F).

### Identification of Somatic Mutations in Tumor Cells

Exome sequencing was performed for tumor cell lines and normal tissue (PBMC) from patients KADA and ANRU. A large number of somatic mutations including single-, double-, and triple-nucleotide variants as well as insertions and deletions were found for both tumor cell lines (Supplementary Table 1). Many of these were found to be non-silent with potential to result in neoepitopes (Table 1). The amino acid sequences encoded by the mutated alleles, plus 9 preceding and tailing residues, were fed into the NetMHCpan 2.8 algorithm to predict HLA-A^*^02:01-binding epitopes allowing for 9- or 10-mers as output. The net result was 1961 candidate neoepitopes for KADA and 366 for ANRU (Table 1) with a predicted affinity of *K*_d_ ≤ 1 μM for HLA-A2^*^02:01. The number of peptides to screen for ability to activate TIL was further restricted by including only peptides that had the highest predicted affinity of *K*_d_ ≤ 50 nM for HLA-A2^*^02:01 and that originated from proteins that are expressed by melanoma cells (mRNA expression RPKM ≥1 in a transcriptome dataset of 7 melanoma cell lines, data not shown). This rendered 181 and 49 peptides that were used to stimulate KADA and ANRU TIL, respectively.

**Table 1:** Selection of mutated peptides predicted to bind HLA-A2 with high affinity from whole exome sequences of tumor cell lines.

**Mutation type**	**Prediction category**	**Patient**	
		**KADA**	**ANRU**
SNV	Non-synon. mutations	2554	323
	Predicted HLA-A2 peptides	1713	182
	Ordered HLA-A2 peptides	165	37
DNV,TNV	Non-synon. mutations	120	9
	Predicted HLA-A2 peptides	37	7
	Ordered HLA-A2 peptides	12	0
InDels	Non-synon. mutations	571	611
	Predicted HLA-A2 peptides	211	177
	Ordered HLA-A2 peptides	4	12
Total	Non-synon. mutations	3244	943
	Predicted HLA-A2 peptides	1961	366
	Ordered HLA-A2 peptides	181	49

*SNV, single-nucleotide variant; DNV, double-nucleotide variant; TNV, triple-nucleotide variant; InDels, insertion/deletions*.

### Recognition of Mutated Neoepitope Peptides by TIL

TIL were screened for induction of IFN-γ secretion against the 181 and 49 selected mutated peptides in pools of either 10 (KADA) or 5 (ANRU), and individual peptides from positive pools were subsequently identified. For KADA, this resulted in the identification of five peptides, containing mutations from genes KDELR2, CCT4, MYLIP, SVIL, and WDR75, which were able to activate autologous TIL (Figure 1B, Table 2). All were 9-mer peptides, except SVIL that was a 10-mer. However, the corresponding 9-mer SVIL peptide, which was excluded due to predicted HLA-A2 binding affinity just below cutoff criteria (Table 2), was ordered later and shown to active TIL even better than the 10-mer (Figure 1C), pointing out the limitation of MHC-binding predictions. For ANRU, three peptides containing mutations that activated autologous TIL were identified (Figure 1E, Table 2). These peptides were 9- and 10-mers containing the same mutation from the gene ETV6 and a 9-mer containing a mutation from the gene NUP210. For both donors, the responses were decreased by blocking HLA-A2 or MHC-I on target cells.

**Table 2:** Analysis of predicted HLA-A2 binding mutated or tumor associated peptides that trigger activation of tumor-infiltrating lymphocytes and/or are detected in pMHC complexes by MS.

**Patient**	**Mutated epitope**	**Mutated position**	**Mutated sequence**	**Wild type sequence**	**Predicted HLA-A2 binding by mutated epitope (nM)**	**Predicted HLA-A2 binding by wild type epitope (nM)**	**Expression of mutant allele**	**Expression of gene compared to β-actin (fold)**	**Detection of mutant epitope on tumor cells by MS**	**Detection of wild type epitope on tumor cells by MS**	**Expression of gene compared to β-actin after IFNγ treatment (fold)**	**Detection of mutant epitope on tumor cells after IFNγ treatment**	**Detection of wild type epitope on tumor cells after IFNγ treatment**
**KADA**	AGPS 9mer^#^	2	ALWDRVVDL	APWDRVVDL	18.12	18335.02	65%	3.28	Yes	No	4.14	Yes	No
	ENC1 9mer^#^	3	YLSELLQTV	YLPELLQTV	2.25	3.1	58%	0.72	Yes	Yes	1.47	Yes	Yes
	KDELR2 9mer	4	ILWIFSIYL	ILWTFSIYL	16.96	4.95	67%	8.07	ND	ND	11.39	ND	ND
	CCT4 9mer	1	FLLDSCTKL	SLLDSCTKL	4.31	23.02v	48%	12.45	Yes	Yes	22.79	Yes	Yes
	MYLIP 9mer	2	RLDAVLMEV	RPDAVLMEV	5.75	6090.18	52%	0.16	No	No	0.36	No	No
	SVIL 9mer	5	YLTDKDFEF	YLTDEDFEF	75.01	156.56	35%	0.62	No	No	0.43	No	No
	SVIL 10mer	5	YLTDKDFEFA	YLTDEDFEFA	13.53	14.11	35%	0.62	No	No	0.43	No	No
	WDR75 9mer	1	FMFVNSLLL	SMFVNSLLL	7.36	62.76	37%	5.18	No	No	9.67	No	No
	Flu M1 9mer	NA^£^	NA	GILGFVFTL	NA	15.03	NA	NA	NA	NA	NA	NA	NA
**ANRU**	ETV6 9mer	1	VLWDYVYQL	LLWDYVYQL	2.24	2.16	55%	0.92	No	No	3.68	Yes	Yes
	ETV6 10mer	1	VLWDYVYQLL	LLWDYVYQLL	4.8	4.34	55%	0.92	ND	ND	3.68	ND	ND
	NUP210 9mer	8	AIDAALTFV	AIDAALTSV	17.01	34.36	42%	1.78	No	No	2.17	No	No
	MART 10mer	2^*^	ELAGIGILTV	EAAGIGILTV	375.16	7627.98	NA	71.4	NA	No	28.91	NA	No
	MART 9mer	NA^¤^	NA	AAGIGILTV	NA	3448.53	NA	71.4	NA	No	28.91	NA	No
	gp100 9mer	2^*^	IMDQVPFSV	ITDQVPFSV	5.47	188.19	NA	591	NA	ND	175	NA	ND

# *Undetected by TIL activation*.

£ *Viral peptide without mutations*.

¤ *Unmutated peptide*.

* *Heteroclitic peptide with optimized aminoacid at mentioned position*.

*NA, Not applicable; ND, Not determined*.

Next, we interrogated whether TIL could distinguish between mutated and corresponding wild-type peptides. Of note, none of the wild-type peptides were able to activate TIL [Figure 1C (KADA) and Figure 1F (ANRU)], even if the predicted binding of the mutated and the wild-type peptide was very similar in most cases (Table 2). The predictions were confirmed by HLA-A2 stabilization assays performed on T2 cells (Supplementary Figure 2). Thus, recognition of neoepitopes is highly specific, and tolerance to wild-type antigens has not been broken. Furthermore, the discrimination between mutated and corresponding wild-type peptides from KADA and ANRU resides mainly on the TCR side of the MHC/peptide/TCR interaction.

### Presentation of Mutated Peptides on Tumor Cell MHC-I by MS

Immune peptidomes for each of the tumor lines were obtained from affinity-purified (W6/32) peptide-HLA class I complexes. Employing Poisson LC-DIAMS, the immune peptidome MS/MS spectra were compared to the fragmentation patterns and elution positions for the 181 and 49 synthetic peptide candidate neoepitopes (Table 1).

**Figure 2:**
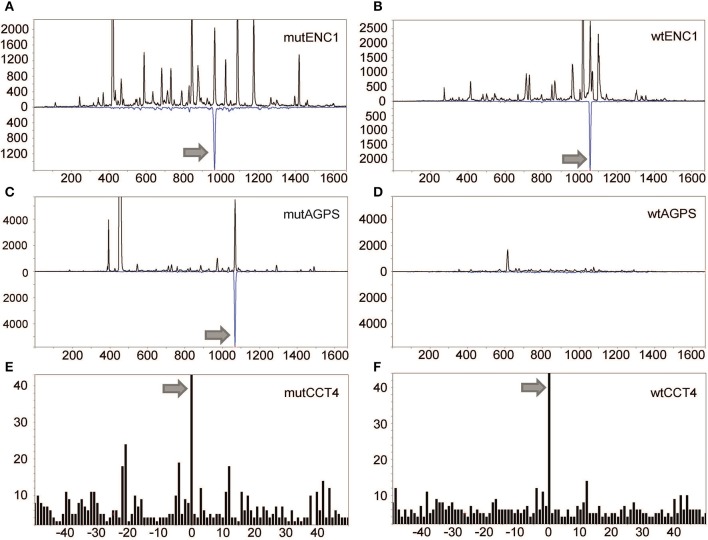
Detection of mutated peptides presented on the tumor cell surface. LC-DIAMS Poisson detection plots for neoepitopes from mutated **(A)** and wild-type **(B)** ENC1 and mutated **(C)** and wild-type **(D)** AGPS from 500,000 cells. Top black traces are extracted ion chromatograms for *m*/*z* of the doubly charged precursor ion in units of counts *per second*. The inverted traces (blue) are Poisson chromatograms showing the number of events, scaled 10-fold (as a convenience in plotting), that can be embedded at fixed cutoff probability in the MS/MS spectrum of the DIA window containing the precursor *m*/*z* (17). Nanospray MS3 Poisson detection of cysteine-containing neoantigen peptide CCT4 **(E)** or the corresponding wild-type peptide **(F)** was performed from 1.5 million cells, as marked with a 0-offset Poisson peak (18).

For KADA, patterns were obtained for 136 of the 181 synthetic peptides with the 45 unobserved peptides either containing cysteine residues (*n* = 23) or being very hydrophobic (*n* = 11) or unobserved for undetermined reasons (*n* = 11). Two peptides from the predicted KADA neoepitopes could be detected (AGPS and ENC1; Figures 2A,C). The cysteine-containing CCT4 neoepitope recognized by TIL was detected using nanospray MS3 with Poisson detection (Figure 2E) (18). The KDELR2 neoepitope recognized by TIL was too hydrophobic for either our LC-MS or nanospray analysis and could not be analyzed. It is noteworthy that corresponding wild-type epitopes could also be detected for ENC1 (Figure 2B) and CCT4 (Figure 2F), but not so for AGPS (Figure 2D), which was calculated to be an HLA-A^*^0201 non-binder (Table 2, Supplementary Figure 2A).

For ANRU, fragmentation patterns were obtained for 38 of the 49 peptides in the ordered library with two of the unobserved peptides being rich for cysteines, seven being very hydrophobic, and two being undetected for unknown reasons. However, none of the candidate neoepitopes, or MART-1 could be detected on ANRU tumor cell MHC-I by MS (data not shown). The ETV6 10-mer was among those epitopes that were too hydrophobic to be detected.

A high-purity isotope-labeled MYLIP peptide was added to the KADA sample to determine if the failure to detect TIL-activating peptides by MS reflected insufficient sensitivity. To address the potential for sample handling losses prior to adding the quantitation peptide, HLA class I complexes were tracked by native Western blots throughout the affinity isolation procedure and shown to be efficiently captured (Supplementary Figure 3A). Spiking 100 attomoles of isotope-labeled mutant MYLIP neoepitope into peptide–HLA complexes from 500,000 KADA cells generated unambiguous Poisson detection (Supplementary Figure 3B) with a peak amplitude of 1,300 counts *per second* (cps). The reference MS and MS/MS spectra for the natural isotope MYLIP neoepitope can be scaled by the ion signal and elution profile and reinserted into the MS data, showing that the MYLIP peptide would be readily detected with only 10 copies (eight attomoles) per cell (Supplementary Figure 3C). This result argues against low sensitivity as a reason for failure to detect this peptide on the KADA tumor cell line. Also, the inability to detect the MART-1 peptide by MS was investigated by quantitative spiking a high purity but unlabeled sample of the native MART-1 9mer, AAGIGILTV. This MART-1 peptide, reported to be naturally processed and presented on HLA-A2, was added at 10 attomoles and gave similar detection sensitivity as the MYLIP peptide (Supplementary Figure 3D). However, AAGIGILTV is not predicted to bind HLA-A^*^02:01 well (Table 2) and has been shown to generate an unstable complex with a short half-life (21). If MART-1 complexes are generated at a high rate, they could activate TIL but may not survive the isolation procedure required for MS analysis.

To confirm that the genes containing the mutated peptides that were recognized by TIL, but not presented on the tumor cells, were indeed expressed by the tumor cells, mRNA levels of each of the genes (total expression of mutated and germline alleles) were compared to β-actin (Table 2). All of the mutated proteins that were recognized by TIL or that were presented by tumor cell MHC-I were clearly expressed. Furthermore, the RNA level of the mutated compared to the germline allele was analyzed, and as expected, both the mutated and the wild-type alleles were expressed (Table 2, Supplementary Table 2). Of note, however, the expression levels of all mutated genes were substantially lower than those of MART-1 and gp100 in ANRU cells, whose levels were more than 71-fold and 591-fold, respectively, higher than that of β-actin.

### Assessment of the Frequency and Avidity of Neoepitope-Specific T Cells in TIL

**Figure 3:**
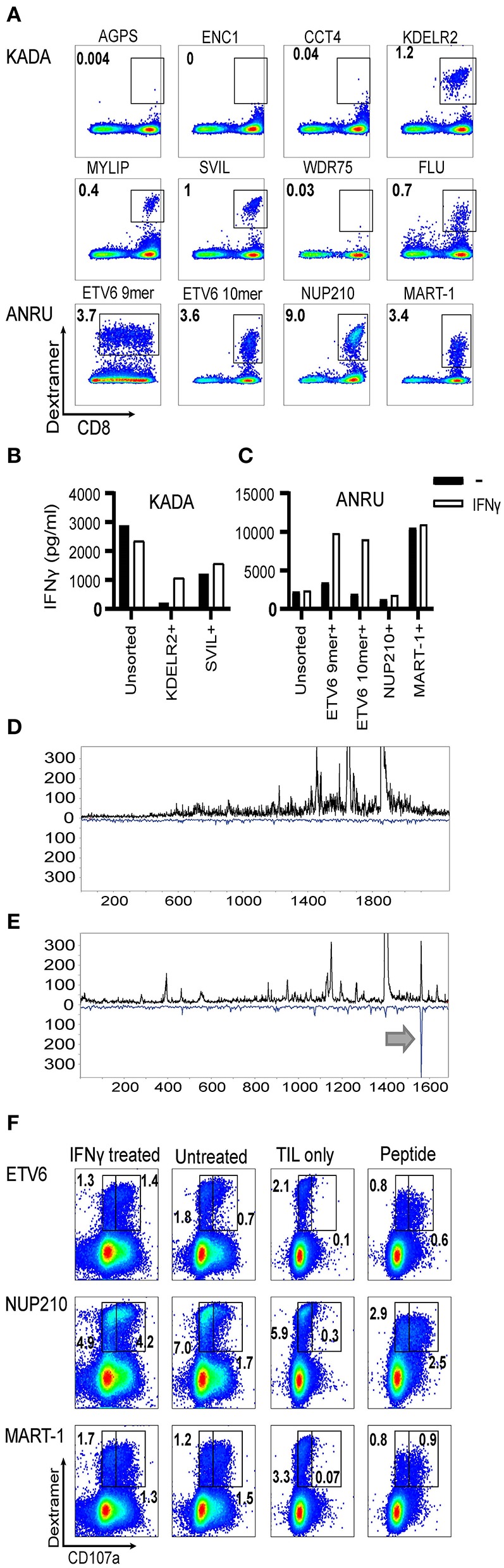
Frequencies of neoantigen-specific T cells in TIL and assessment of their ability to recognize autologous tumor cells. HLA-A2/mutated peptide dextramers were produced for peptides found to activate TIL and/or found to be presented on tumor cell MHC-I. The dextramers were used to stain tumor-infiltrating lymphocytes **(A)**, and anti-PE beads were used to enrich for stained cells followed by a rapid-expansion protocol. Thereafter, recognition of KADA **(B)** and ANRU **(C)** tumor cells by the sorted TIL was assessed by IFN-γ ELISA with or without IFN-γ pretreatment of the tumor. Only the specificities that could be significantly enriched by the sorting are shown. Unsorted cells were used as a control. The tumor cells were analyzed for neoepitope expression by MS and presented as LC-DIAMS Poisson detection plots for mutant ETV6 in untreated **(D)** and IFN-γ-treated ANRU tumor cells **(E)** with the arrow in the latter indicating detection. The total TIL population (**F**. ANRU) was co-cultured with untreated or IFN-γ pretreated autologous tumor cells, or with the different neoepitope peptides, analyzed by dextramer staining for the same epitope and for cell surface CD107a. As negative control, TIL alone were used and stained as described above. Dot plots are gated on lymphocytes/singlets/live cells and frequency indicates % dextramer^+^ out of CD8^+^ cells **(A)** and gated in the same way plus on CD8^+^ and frequency indicates % dextramer^+^CD07a^−^ or dextramer^+^CD107a^+^ of CD8^+^ cells **(F)**.

To detect and measure the frequency of neoepitope-specific TIL within the total TIL population, neoepitope-specific PE-conjugated peptide/MHC dextramers were custom ordered. In KADA TIL (Figure 3A), stained populations could clearly be detected for KDLER2, MYLIP, and SVIL epitopes. Staining with CCT4 dextramers was very weak and, for WDR75 dextramers, virtually absent. Furthermore, dextramers were produced also for AGPS and ENC1, the neoepitopes detected on KADA cell MHC-I exclusively by MS. In line with the TIL stimulation results, the AGPS dextramer did not bind TIL at all, while ENC1 showed a weak level of staining, impossible to separate from background staining. In ANRU TIL, on the other hand (Figure 3A), all the custom dextramer stainings (ETV6 9- and 10-mer, NUP210) resulted in well-defined populations, comparable to the staining seen with the MART-1 dextramer (Supplementary Figure 1A).

The weak CCT4 dextramer staining indicated that the interaction of the specific T cells with the MHC/peptide complex was of lower avidity. In addition, functional avidity of the specific T cells was assessed by titrating the peptides directly onto TIL and measuring the activation as IFN-γ secretion [Supplementary Figure 3E (ANRU) and Supplementary Figure 3F (KADA) and Supplementary Table 3]. These titration curves indicate that the activation of specific T cells by MHC-bound CCT4, KDLR2, SVIL 10-mer, ETV6 10-mer, and wild-type MART-1 peptides is of lower functional avidity that requires micromolar concentrations of peptide for activation.

### Ability of Neoepitope-Specific T Cells to Recognize Autologous Tumor Cells

We next aimed to test the capacity of TIL-derived neoepitope-specific T cells to recognize the autologous tumors from which they were derived. We first attempted to enrich neoepitope-specific T cells from TIL by sorting neoepitope dextramer-stained TIL using anti-PE-coupled magnetic beads. The selected cells were subjected to a round of rapid expansion, as were unsorted TIL as a control. For KADA, only the population specific for KDELR2 and SVIL epitope could be effectively enriched by this approach (79 and 13% dextramer-stained cells in the sorted TIL vs. 1.1 and 0.3% in the unsorted, respectively). Although some enrichment was achieved also for the other neoepitope-specific T cell populations from KADA, the enriched TIL populations still contained a high proportion of dextramer-negative cells, probably due to the low starting frequency of the dextramer-targeted TIL (<0.2%). For ANRU, enrichment could be performed for all neoepitope dextramers with 16–34% (NUP210:28%, ETV6 9-mer: 16%, ETV6 10-mer: 34%) dextramer-stained cells in sorted TIL vs. 2.1–4.8% (NUP210: 5.1%, ETV6 9-mer: 2.2%, ETV6 10-mer: 2.3%) in unsorted TIL. Sorted TIL were co-cultured with autologous tumor cells and analyzed for tumor recognition. All enriched neoepitope-specific T cells were also functionally enriched as they recognized their corresponding peptide with increased efficiency (data not shown). However, disappointingly, none of the enriched neoepitope-specific populations recognized tumor cells better than unsorted TIL [Figure 3B (KADA) and Figure 3C (ANRU)]. These results were in line with the MS results. However, MART-1-specific TIL were successfully enriched (63% dextramer-stained cells in sorted TIL vs. 3.6% in unsorted TIL) and recognized the autologous tumor better than unsorted TIL (Figure 3C). Nevertheless, the MART-1 epitope was not detected by MS on tumor MHC-I. The reason for this may be the short half-life of the MART-1/MHC complex as already discussed.

### Analysis of Neoepitope Presentation on MHC-I and Activation of Specific TIL After IFN-γ Treatment of Tumor Cells

We next asked if the lack of neoepitope presentation by tumor MHC-I and the concurrent inability of neoepitope-specific TIL to recognize these cells could be due to an inefficient peptide processing and presentation machinery. IFN-γ-treated KADA and ANRU tumor cells showed markedly increased expression of MHC-I surface antigens by FACS [Supplementary Figure 4A (KADA) and Supplementary Figure 4B (ANRU)], and components of the peptide presentation machinery (APM) by Western blot (TAP1 and 2, Tapasin, MHC-I heavy chain, LMP2 and 10 and β2m; Supplementary Figure 4C) and RT-qPCR (data not shown). Therefore, we investigated if pre-treatment of tumor cells with IFN-γ would affect the expression of the mutated genes or the presentation of the neoepitopes on MHC-I. The mRNA expression levels in untreated vs. IFN-γ-treated cells did not display any dramatic upregulation of any of the neoepitopes (Table 2) but interestingly there was a clear decrease of both MART-1 and gp100 expression after IFN-γ treatment. Although most of the neoepitopes were still not detected by MS, the 9-mer ETV6, which was not present on untreated ANRU cells (Figure 3D), could be detected by MS analysis on ANRU tumor cell MHC-I after IFN-γ treatment (Figure 3E). The same cytokine-inducible upregulation was true for the ETV6 9-mer wild-type peptide (Table 2). The ETV6 10-mer could, as mentioned before, not be analyzed by MS due to their hydrophobic nature.

Next, the ability of neoepitope-specific TIL to recognize IFN-γ-treated tumor cells was assessed. This was first interrogated using the dextramer-sorted neoepitope-specific TIL populations. For KADA, there was an increased recognition of IFN-γ-treated tumor cells compared to untreated tumor cells by TIL enriched by KDELR2 dextramers (Figure 3B), which, as mentioned, could not be analyzed by MS due to its hydrophobicity. For ANRU, in line with the MS results, the same was true for TIL enriched by ETV6 9- or 10-mer dextramers, while the MART-1-sorted TIL recognized untreated and IFN-γ-treated tumor cells to the same high extent (Figure 3C). Since the sorting of neoepitope specific TIL was less efficient for several epitopes, we next investigated the recognition of IFN-γ-treated tumor cells starting from the total TIL population. Unsorted ANRU TIL, which has a high frequency of neoepitope specific T cells, were co-cultured with IFN-γ-treated and -untreated tumor cells (Figure 3A). Neoepitope-specific T cells were identified with dextramers after co-culture. The activation was analyzed as degranulation by measuring CD107a surface expression and by comparing the brightness of the dextramer staining, since activation is known to lead to decreased levels of the TCR on the cell surface. In line with the MS-results, ETV6 dextramer-stained cells expressed CD107a and displayed decreased levels of dextramer-staining when cultured with IFN-γ-treated tumor cells (Figure 3F). In addition, and in contrast to both the MS results and the results with the dextramer-sorted cells, in this experiment, NUP210-dextramer-positive cells recognized the tumor demonstrated by tumor-induced activation of degranulation (Figure 3F).

To better characterize the relation between MS sensitivity and TIL response, IFN-γ-treated ANRU cells were loaded with NUP210 peptide at a lower concentration than was required to activate IFN-γ production by TIL (Supplementary Figures 3G,H). MS sensitivity was substantially greater than biological assay as detailed therein.

### Generation of Neoepitope-Specific T Cells From Blood

**Figure 4:**
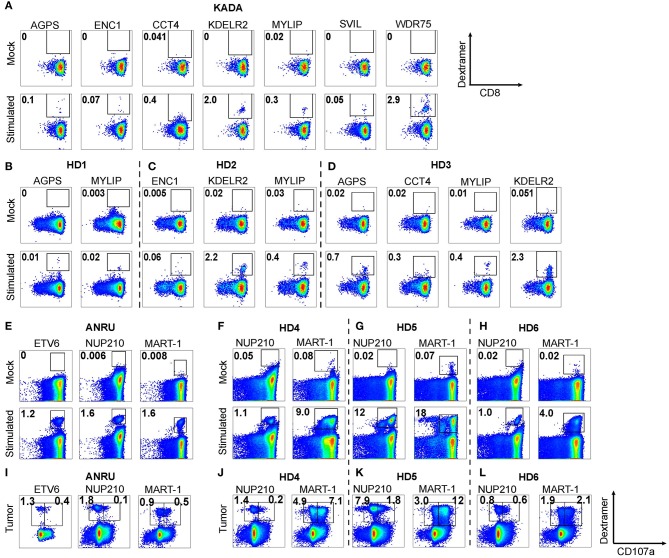
Expansion of neoepitope peptide-specific T cells from PBMC. Dendritic cells from KADA **(A)** or ANRU **(E)** were loaded with their respective TIL-activating and/or tumor-presented peptides and used to stimulate autologous CD8 T cells. The same experiment was performed using three healthy donors for each peptide set (**B–D**, KADA neoepitopes; **F–H**, ANRU neoepitopes and MART-1). After 10 days of expansion, the cells were stained with corresponding HLA-A2/peptide dextramers and analyzed by flow cytometry. Dot plots are gated on lymphocytes/singlets/live cells and frequency indicates %dextramer^+^ out of CD8^+^ cells. For ANRU epitopes, the function of the neoepitope specific T cells was assessed by re-stimulation with ANRU tumor cells and evaluated by CD107a staining (**I**, ANRU; **J–L**, healthy donors). Only positive stainings are shown. Dot plots are gated on lymphocytes/singlets/live cells/CD8^+^ cells and frequency indicates % dextramer^+^CD07a^−^ or dextramer^+^CD107a^+^ of CD8^+^ cells.

Since we found two neoepitopes, from mutated AGPS and ENC1, that were expressed on ANRU tumor MHC-I, but that were not recognized by TIL, we wanted to determine whether these epitopes were selectively non-immunogenic for the patient, broadly non-immunogenic, or, alternatively, if we could expand neoepitope-specific T cells from healthy donors. To test these possibilities, CD8+ T cells derived from blood from patient KADA (Figure 4A) or three healthy HLA-A2+ donors (Figures 4B–D) or from patient ANRU (Figure 4E) and three other HLA-A2+ healthy donors (Figures 4F–H) were stimulated with autologous monocyte-derived DC pulsed with peptides. The CD8+ T cells were screened for ability to recognize each peptide that had either been found to activate TIL or to be presented on the tumor cell MHC-I. Of note, for KADA, all of the peptide-stimulated PBMC cultures had detectable neoepitope specific CD8+ T cells, shown by dextramer-positive populations. For KDELR2 and WDR75, a high frequency of strongly stained cells was detected, and for AGPS, ENC1, MYLIP, and SVIL, the staining was strong and distinct, although the frequencies of stained cells were low. For CCT4, however, few cells were stained and the staining was also weak, suggesting that cells had expanded but were of lower affinity, similarly to the situation in KADA TIL. In addition, in the healthy donors, most of the neoepitope-specific T cell populations could be expanded in at least one donor, except for WDR75 (Figures 4B–D and data not shown). In contrast, in mock stimulated (unpulsed DC) cultures, there was no staining with any of the dextramers for any of the individuals. For ANRU, all the peptides expanded specific cells in ANRU PBMC, resulting in clearly defined dextramer-stained populations. In all three healthy donors, both NUP210 and MART-1 stimulations resulted in clear dextramer-positive populations (Figures 4F–H).

Thus, all the mutated peptides were found to be immunogenic, even for T cells derived from the blood of both donors (KADA and ANRU) from which the autologous tumor cell lines were derived. Importantly, the blood was drawn from these patients long after the tumor was removed, and they were both cancer free at the time. Therefore, it is not surprising that there were no tumor-specific cells found in mock-stimulated blood. Of particular importance, however, even the MS-defined neoepitopes that were unable to activate the autologous KADA TIL (AGPS and ENC1) could expand T cells from both KADA PBMC and from HLA-A2+ donor PBMC.

We also investigated if the neoepitope specific CD8+ T cells, derived from ANRU PBMC or from the three healthy HLA-A2+ donors, could recognize the ANRU tumor cells. Therefore, the stimulated CD8+ (DC pulsed with peptides) were re-stimulated with ANRU tumor cells and recognition was measured by FACS as increased CD107a expression/degranulation [Figure 4I (ANRU) and Figures 4J–L (healthy donors)]. A portion of the ANRU neoepitope and MART-1-specific CD8+ T cells recognized the autologous tumor observed by dextramer and CD107a double-positive cells. All three healthy donors' CD8+ T cells stimulated either with DC pulsed with the NUP210 or the MART-1 epitope could also recognize the ANRU tumor. As a positive control, for each neoepitope, stimulated CD8+ cells were re-stimulated with the peptide, which resulted in activation of CD8+ T cells for each epitope (data not shown).

## Discussion

Infiltrating CD8+ T cells mediate the predominant immune response in malignant melanoma (22). Accordingly, infusion of in vitro-expanded TIL which are dominated by CD8+ T cells commonly produces complete and long-lasting regressions of metastatic lesions (1). There is, however, limited information on the precise specificity of the tumor-derived T cells that mediate tumor rejection in melanoma patients, and the extent to which important tumor epitopes are derived from broadly expressed shared tumor antigens or from private mutated tumor epitopes. We followed two parallel but distinct strategies to discover immunogenic neoeptitopes: one based on peptide reactivity of T cells isolated from the patient's tumor, the other on physical detection of putative neoepitopes presented on the surface of the tumor cells.

We performed whole exome sequencing of early passage tumor cell lines originated from two stage III/IV metastatic melanoma patients, to identify mutated epitopes based on *in silico* predicted HLA-A2 binding and expected expression in melanoma. Similar to others (23), we focused on MHC-I HLA-A02:01 restricted epitopes, motivated by more developed and precise algorithms for predicting T-cell epitopes for MHC-I and in particular for HLA-A02:01. In addition, Swedish patients with advanced melanoma have a higher prevalence for HLA-A02:01, in particular in those patients with a poor prognosis (24).

The TIL were derived from two long-term survivors (KADA and ANRU), indicating an ongoing immune response. Their TIL demonstrated strong reactivity against autologous tumor lines, providing us with an efficient tool for screening the predicted neoepitope library, in a similar fashion to what has been performed before by others (23). This resulted in the identification of a total of eight neoantigen epitopes (five from KADA and three from ANRU) with the ability to activate IFN-γ release and degranulation by autologous TIL. The finding that only 8 out of 230 mutated neoepitopes predicted to bind MHC-I were recognized by the patients' TIL confirms similar observations from others (23, 25).

One might expect that the presence in TIL of T cell clones specific for these eight epitopes, all originating from proteins that were highly expressed at the mRNA level in the tumor cells, would signify that those same epitopes should be presented on MHC-I on the surface of the tumor cells. This was, however, not the case, as shown by MS analysis of those peptides that were actually presented in association with MHC-I on tumor cells. In fact, the 10,000-fold greater rate of cellular protein turnover vs. pMHC turnover mandates that few, if any, peptides derived from a given protein are expressed on the cell surface complexed to MHC-I (26).

Conventionally, discrepancies between T cell recognition and MS detection are attributed to poor MS sensitivity. The lack of false negatives associated with the data-independent acquisition format (see Figures 2, 3 and Supplementary Figure 3), the tracking of MHC-I complexes during the affinity isolation, the calibration of MS sensitivity into the single attomole (10^−18^ mole) level by the addition of internal standards, and the recovery and MS detection peptides, loaded onto tumor cells at concentrations below TIL recognition, are however hard to reconcile with neoepitope detection failing due to MS sensitivity. This said, peptide–MHC-I complex instability and the reduced sensitivity with cysteine-containing or very hydrophobic peptides are limitations of our current MS methodology. The approach of identifying peptides that activate T cells by functional assays and in parallel detecting peptides presented on tumor cell MHC-I by MS provided us with a unique methodological comparison. Several important conclusions can be drawn from this comparison, pointing at strengths and limitations of each of these two methods.

First, we can conclude that our MS approach allows detection of three HLA-A2 restricted neoepitopes presented on the melanoma cells (AGPS, ENC1, CCT4; Table 2). For one of these (CCT4 from the KADA tumor), we were able to show recognition by the autologous TIL and expansion of specific T cells from patient peripheral blood. In spite of this, we were unable to specifically sort out the T cell clones recognizing the neoepitope CCT4, most likely explained by poor binding to the peptide/MHC dextramer resulting in difficulties in enriching this T cell population, leaving us with no possibility to prove the expected tumor recognition by these CCT4 specific T cells.

For the two other epitopes identified by MS as presented on KADA tumor MHC-I (AGPS, ENC1), we could unequivocally demonstrate that these epitopes were not recognized by the autologous TIL. Since these epitopes were demonstrated to be immunogenic after culturing PBMC with peptide-loaded DCs, one explanation for the absence of reactivity is a low level of antigen. We expect that DC cross-presentation of phagocytosed cellular debris cannot display a substantial fraction of MHC-I-binding peptides from the tumor's full proteome and the likelihood of cross-presenting DC activating neoepitope-specific CD8 T cells decreases as the abundance of the mutant source protein decreases. When abundance is under the threshold for priming and expansion by cross-presenting DCs in draining lymphoid tissues, the full repertoire of circulating and lymphoid-resident T cells may not be activated, expanded, and deployed for neoepitope recognition. Lack of epitope recognition may also reflect immunodominance, where immune responses target only a few antigenic peptides of the many displayed, thereby curtailing natural responses against non-dominant epitopes and/or be a consequence of ineffective cross-presentation of the epitopes (27–29). There is no obvious dominance of KADA TIL responses, however. Peripheral tolerance mechanisms mediated by MDSC and regulatory T cells in melanoma patients may also limit the ability of TIL to respond to these antigens (30). Heterogeneity of neoantigen expression, resulting in T cells reactive with individual, sub-clonal mutations (presence in only a subset of tumor cells) and not with clonal mutations (presence in all tumor cells) may also explain the absence of reactivity against certain epitopes (31).

Immunogenicity of a neoepitope has been reported to arise both due to changes in anchor residues (32) and TCR contacts (33). In our study, the majority of neoepitopes that could activate TIL had not gained binding affinity to HLA-A2 compared to the corresponding wild-type peptide; only for the AGPS and MYLIP peptides from the KADA tumor was an increased binding observed, as confirmed by HLA-A2 stabilization assays. Also, the corresponding wild-type epitope could not activate TIL, indicating that tolerance to the wild-type antigen has not been broken. These findings are therefore in line with those of Fritsch and collaborators (34), who found mutations located in the TCR-facing residues of the neoepitopes rather than the anchor residues when analyzing 40 neoepitopes of human cancers that induced immune responses associated with regression or long-term disease stability.

As expected (15), IFN-γ resulted in increased levels of MHC-I on the tumor surface and of various components of the MHC-I APM. In addition, MHC presentation of a neoepitope derived from the protein ETV6 was detected by MS in IFN-γ-treated, but not in untreated ANRU tumor cells. This may be explained either by the observed total increase in MHC-I, or alternatively and more likely by an altered peptide repertoire induced by IFN-γ-mediated increase in the expression of the immune proteasome (LMP2 and 10) which we found to have a markedly enhanced expression. In line with the ETV6 peptide being presented, we could also detect an enhanced recognition of the tumor cells by dextramer-sorted TIL specific for this peptide compared to unsorted TIL. Such a cytokine-inducible epitope display is consistent with the importance of an intact antigen presentation and IFN-γ signature for immunotherapy to be efficient (35). Also, IFN-γ-treated KADA tumor cells, but not the untreated tumor cells, showed increased stimulation of TIL enriched for T cells specific for the KDELR2 epitope. We were, however, unable to confirm with MS the MHC-I presentation of the KDELR2 epitope on IFN-γ-treated tumor cells due to the hydrophobic nature of this epitope. IFN-γ-treated tumor cells were also recognized by TIL specific for the neoepitope NUP210, but the activation only resulted in degranulation, not in IFN-γ production.

**Figure 5:**
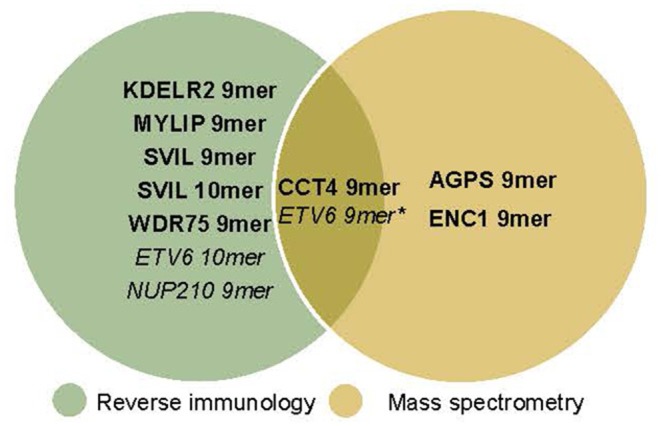
Neoepitopes detected by reverse immunology vs. mass spectrometry. With reverse immunology TIL reactivity against 5/181 (**KADA**) and 3/49 (*ANRU*) of the predicted neoepitopes were observed. With mass spectrometry 3/136 (**KADA**, not all peptides could be analyzed) and 1*/38 (*ANRU*, not all peptides could be analyzed) of the predicted neoepitopes were detected on the cell surface. **KADA** epitopes are in Bold, *ANRU* epitopes are in Italic. *Detected after IFNγ treatment of the tumor.

Several other possible explanations may account for the discrepancies between TIL recognition of tumor cells and MS detection of the peptides on tumor MHC-I, which are summarized in Figure 5. A better understanding of the basis of these divergent results are essential for future developments of optimal methods for clinical application. Since TIL were blocked by MHC-I antibody, our data argue strongly against non-specific activity and favor the interpretation that TIL are activated by an epitope/MHC-I complex. One possibility is that part of the results could be explained by cross-reactivity of TCR on the TIL with unidentified peptides on the surface of the tumor cells. The high concentrations (μM) of peptides used for screening in the absence of external bioforces that occur *in vivo* and normally tune TCR recognition, fostering antigen discrimination and low copy number pMHC activation of T cells, encourage such TCR cross-reactivity (36, 37).

Another likely explanation for discrepancies between TIL screening result and the MS method lies in limitation in terms of sensitivity for our MS method for known physicochemical challenging peptides and very weak binders. This limitation is likely to explain our difficulties to detect by MS two of the TIL detected epitopes (KDELR2 and MART-1), and limits the usefulness of the MS detection method for such peptides. That said, those outliers are readily flagged as problematic prior to MS analysis. MART-1 is of particular interest, since this epitope is extensively used as a prototype for tumor antigen, also in clinical trials with TCR-modified T cells (38). While MART-1 transcriptional rates are extremely high, the wtMART-1 peptide has orders of magnitude lower affinity for HLA-A2 than the heteroclitic MART-1 counterpart and an unusual TCR binding to MART-1 peptide/HLA-A2 complexes has been documented (39). The latter will foster TCR cross-reactivity, likely also accounting for discordance in TAA heteroclitic MART-1 binding to TIL and their stimulation in the absence of MS detection of native MART-1 on the ANRU tumor cells.

The advantage of the MS approach is that a positive identification can give unequivocal proof for the MHC-I presentation on the patient's tumor, even when the patient's TIL are not available or are not reactive. This is exemplified by the AGPS and ENC1 neoepitopes that were detected by MS as being presented on the tumor lines. When analyzed for immunogenicity by stimulating T cells from PBMC with autologous monocyte-derived DC pulsed with peptide, our results clearly showed that specific T cell populations could be expanded from patient (KADA and ANRU) or normal donor PBMC using autologous monocyte-derived DC. In addition, a population of these specific T cells could recognize and respond with degranulation upon re-stimulation. That only a part of the expanded cells responded could be due to lower affinity of neoepitope-specific T cells derived from blood (40, 41). Notwithstanding, these findings have important therapeutic implications, and clinical applications where MS detected neoepitope-specific T cells are expanded from blood by either *in vitro* stimulation or by vaccination hold great promise for clinical developments. In our ongoing clinical trial (NCT01946373), we are applying a combination of ACT with TIL and a tumor vaccine composed of autologous tumor lysate pulsed monocytic DCs. The results above have motivated us to consider extending our clinical trial to involve ACT with autologous TIL or peripheral blood enriched for neoepitope-specific T cells, followed by a boost of this neoepitope-specific response by a DC tumor vaccine derived from the same mutated epitopes. This type of approach has recently been spurred by results from others, demonstrating T cell activation following administration of DC-, peptide-, or RNA-based tumor antigens to cancer patients (42, 43).

## Data Availability Statement

All datasets generated for this study are included in the article/Supplementary Material.

## Ethics Statement

The studies involving human participants were reviewed and approved by Regionala etikprövingsnämnden i Stockholm, Sweden. The patients/participants provided their written informed consent to participate in this study.

## Author Contributions

SW and TL designed the experiments involving neoantigen and tumor cell line recognition by TIL. BR and ER designed the experiments regarding analysis using mass spectrometry. BS has designed experiments regarding analysis of APM components. MV, IP, and RO planned and coordinated next-generation sequencing. MV performed NGS data analysis and prediction of neoepitopes. SW, TL, MV, BR, JD-C, LH, JR, AM, JM, and RM has performed laboratory work, generated data, and performed data analysis. ML and JH have been involved in discussions. JH recruited the patients. SW, TL, BR, ER, and RK writing of the manuscript. ER and RK have supervised the study.

### Conflict of Interest

RK is a board member/consultant for Clinical Laser Thermia Systems AB and is also a Scientific Advisor for Anocca AB and Phion Pharmaceutics and receives research grants from these 2 companies. He has also been paid for organizing courses in Immunotherapy for BMS Sweden. The remaining authors declare that the research was conducted in the absence of any commercial or financial relationships that could be construed as a potential conflict of interest.
