# Supplementary material for: Cancer Neoepitopes for Immunotherapy: Discordance Between Tumor-Infiltrating T Cell Reactivity and Tumor MHC Peptidome Display
Source: Front Immunol. 2019 Dec 11;10:2766. doi: 10.3389/fimmu.2019.02766 (PMC6918724; doi:10.3389/fimmu.2019.02766)
Supplement: Supplementary file 1 [file Presentation_1.pdf]

Supplementary Information for

Cancer neoepitopes for immunotherapy: discordance between tumor-infiltrating T cell reactivity and tumor MHC peptidome display.

This PDF file includes:

Supplementary text

Figures S1 to S5

Tables S1 to S4

SI References

Supplementary Information Text

Materials and Methods.

Exome sequencing data analysis

Genomic DNA was extracted from tumor cell lines and TIL cultures (germline control) using the QIAamp DNA Mini kit (Qiagen, Hilden, Germany) and submitted to the High Throughput Sequencing unit of the DKFZ Genomics core facility for exome enrichment and sequencing. Exomes were enriched using the SureSelectXT Human v5 kit (without UTRs; Agilent Technologies, Inc.; Santa Clara, CA) and sequencing was performed on a HiSeq2000 machine (Illumina, Inc.; San Diego, CA) in 100bp paired-end mode. For ANRU, 52.4 million germline reads and 125.4 million tumor cell line reads were generated. For KADA, 49.7 million reads covering the germline and 98 million reads from the tumor cell line were produced. Sequence data analysis was performed on the Heidelberg Unix Sequence Analysis Resources (HUSAR) server. For initial mapping of raw reads to the human genome (hg19), the Burrows-Wheeler aligner was used (1). SAM-to-BAM conversion, addition of read groups and marking of duplicate reads were done with Picard (http://broadinstitute.github.io/picard). Re-alignment around InDels and base recalibration were performed with GATK tools with options and settings based on a pipeline published by O'Rawe *et al.* (2). For detection of somatic point mutations (single nucleotide variants, SNVs) we used MuTect (3), while InDel calling was done with VarScan2 (4). For annotation of identified variants we used ANNOVAR (5). Di- and trinucleotide variants (DNVs, TNVs) were identified and the encoded mutations were annotated manually from MuTect output.
We used seq2HLA (6, 7) to resolve the HLA alleles from the respective germline exome data, confirming the previously determined HLA-A*02:01 genotype of both samples. Potential HLA-A*02:01-presented epitopes were predicted from the annotated amino acid sequences by the NetMHCpan 2.8 algorithm (8, 9), allowing 9-, and 10-mers as output.

Isolation of peptide-MHC I complexes from melanoma cell lines

Adherent melanoma cells were released non-enzymatically to preserve surface antigen expression by incubation at 37°C for 30 min in non-enzymatic cell dissociation medium (PBS supplemented with EDTA [5 mM], EGTA [5 mM] and FCS [1%]). Cells were resuspended in PBS (4°C) and adjusted to ~0.5 - 1 × 10^6/mL, a concentration range previously established as optimal for this protocol. For peptide pulsing experiments, NUP210 mutant nonamer peptide (JPT Peptide Technologies) was added to IFNγ-treated ANRU cells to a final concentration of 5 nM, 50 nM or 100 nM in 1 ml non-enzymatic cell dissociation medium. Cells were gently rotated at 37°C for 30 min (5 nM, 50 nM) or 1 h (100 nM).
Subsequently, all cell procedures were carried out under sterile, high efficiency particulate air (HEPA)-filtered conditions at 4°C using low-protein binding tubes (Eppendorf). Following centrifugation at 900g for 5 min, supernatant was removed and the pellet (0.5 – 1 × 10^6 cells) resuspended in 0.4 mL

lysis buffer (Triton X-100, 1.2%; octyl-β-D-glucopyranoside, 1.77%; Tris-HCl, 20 mM; EDTA, 1 mM; NaCl; 100 mM; complete protease inhibitor cocktail, ×1; Roche) and incubated on ice with occasional flicking for 20 min. Lysed cells were centrifuged for 10 min at 21,000 g and the pellet retained for subsequent analysis of MHC I by Western blotting of transfers from native Tris-glycine PAGE gels. The supernatant lysate was centrifuged for 1 h at 105,000 g (Airfuge; Beckman-Coulter) and 4°C to pellet cellular debris. During this centrifugation, Protein G Sepharose beads (20 µL of 50% suspension in PBS; GE Healthcare) were preincubated with anti-monomorphic MHC I antibody (4 µg in 8 µL PBS; clone W6/32; Biolegend). Following ultracentrifugation of the lysate, a sample (25 µL) was removed for subsequent detection of MHC I, as described above. The remaining lysate was added to the W6/32 pre-incubated beads and rotated for 3 h at 4°C. The lysate-bead suspension was centrifuged at 900 g for 3 min and the supernatants retained for confirmation of depletion of MHC I. The MHC I-loaded beads were then washed ×6 in wash buffer (0.5 mL/wash; octyl-β-D-glucopyranoside, 1.77%; Tris-HCl, 20 mM; EDTA, 1 mM; NaCl; 100 mM) followed by ×2 washes in salt buffer (Tris-HCl, 20 mM; EDTA, 1 mM; NaCl; 100 mM). Residual liquid was removed using fine pipette tips and the bead samples stored at -80°C until processed for MS analysis.

Supplementary fig 1

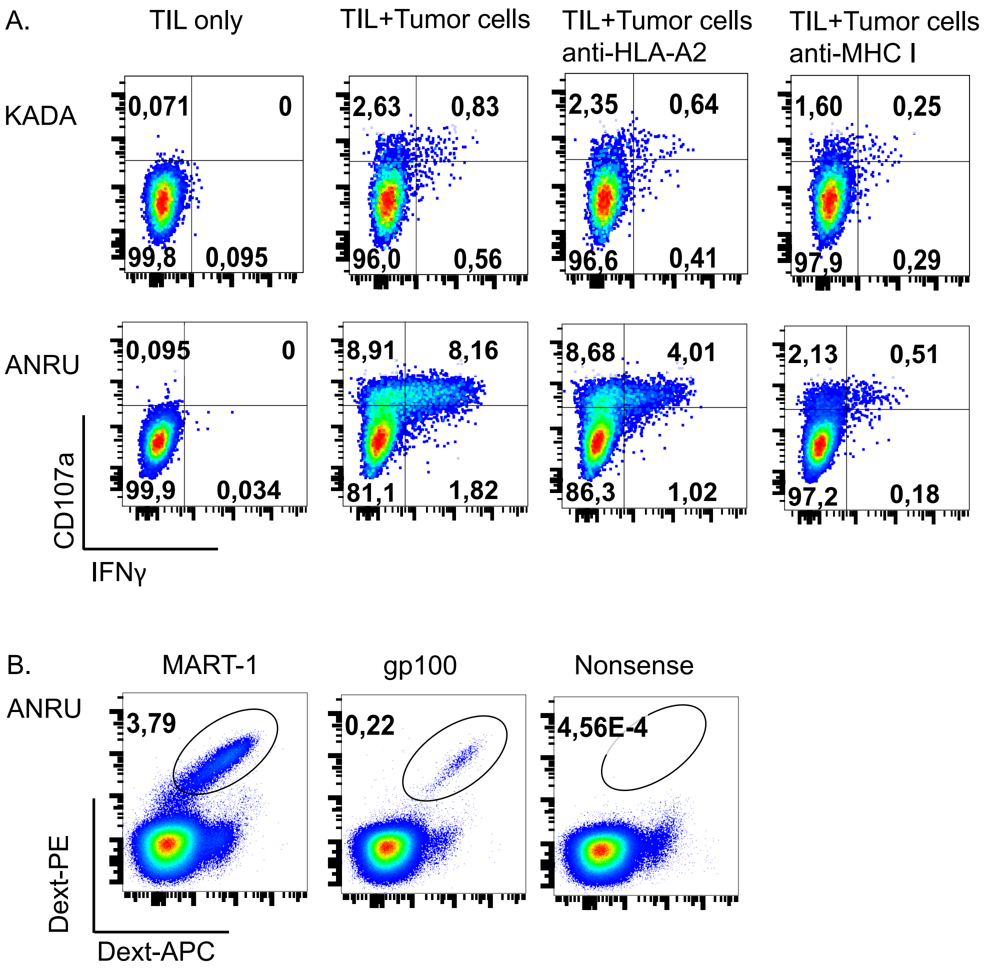

S1. Recognition of autologous tumor cells and common tumor-associated antigens by tumor-infiltrating lymphocytes. The ability of TIL to recognize autologous tumor cells was assessed in co-cultures, with or without HLA-A2 or MHC class I blocking-antibodies, and degranulation (CD107a) and IFN-γ production were analyzed after 6h by intracellular cytokine staining and flowcytometry (A). Recognition of common tumor-associated antigens by TIL was assessed by dextramer staining (B. ANRU, positive dextramers and a negative control are shown). No dextramer binding was detected for KADA. All dotplots are gated on Lymphocytes/Singlets/Live cells/CD3+ cells/CD8+ cells.

Supplementary fig. 2

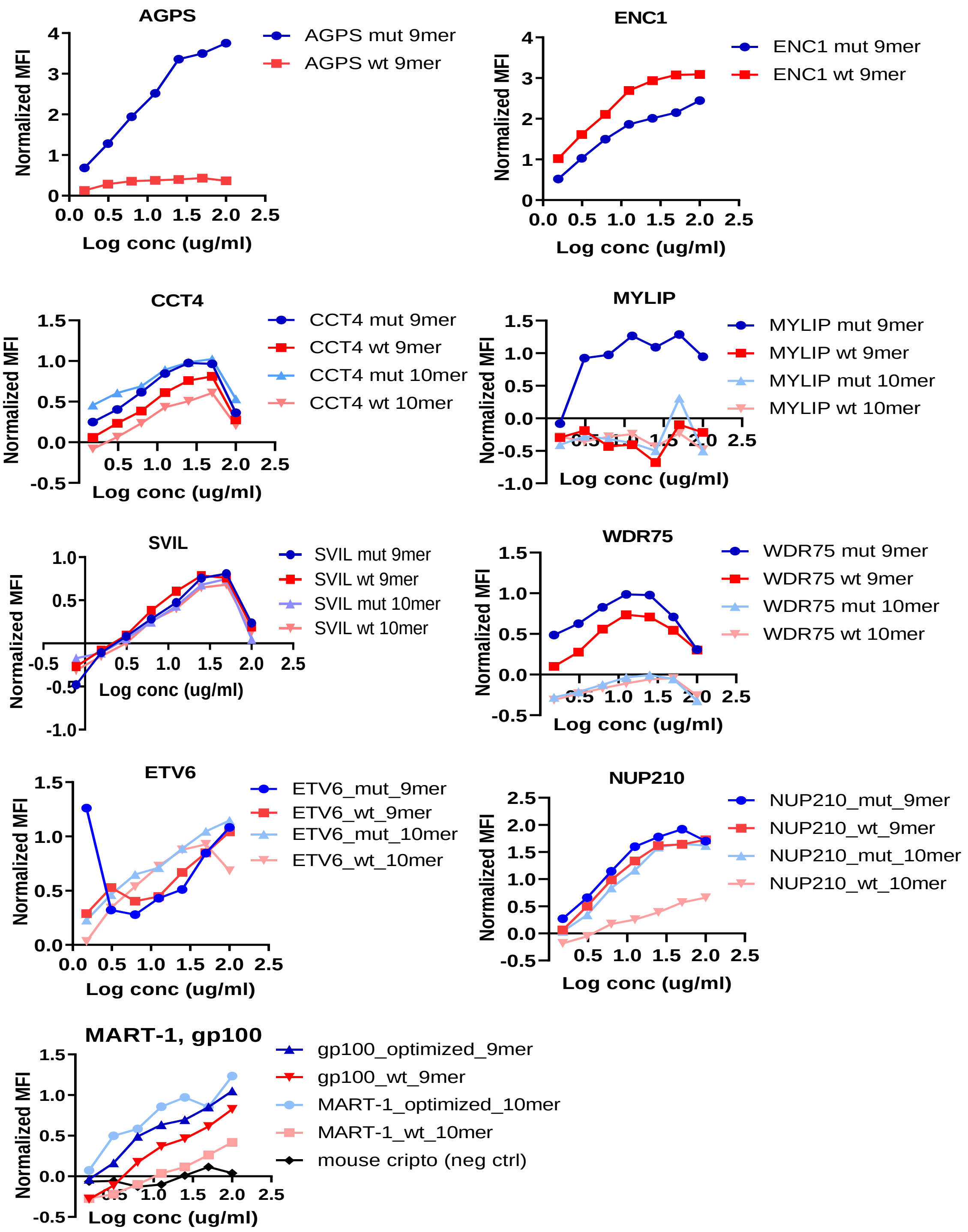

S2. *Determining binding affinities of neoepitope peptides to HLA-A2.* Binding affinities of mutant and wild type AGPS (A), ENC1 (B), CCT4 (C), MYLIP (D), SVIL (E), WDR75 (F), ETV6 (G), NUP210 (H), and control peptides for optimized or native MART-1 and gp100 or negative control mouse cripto (I) peptides were determined by a HLA-A*02 stabilization assay using T2 cells. Binding affinity was assessed by a HLA-A*02 staining that was analyzed by flow cytometry. Geometric mean fluorescence intensities (gMFI) were normalized according to the formula: (gMFI (peptide)-gMFI (no peptide)/gMFI).

Supplemental fig. 3

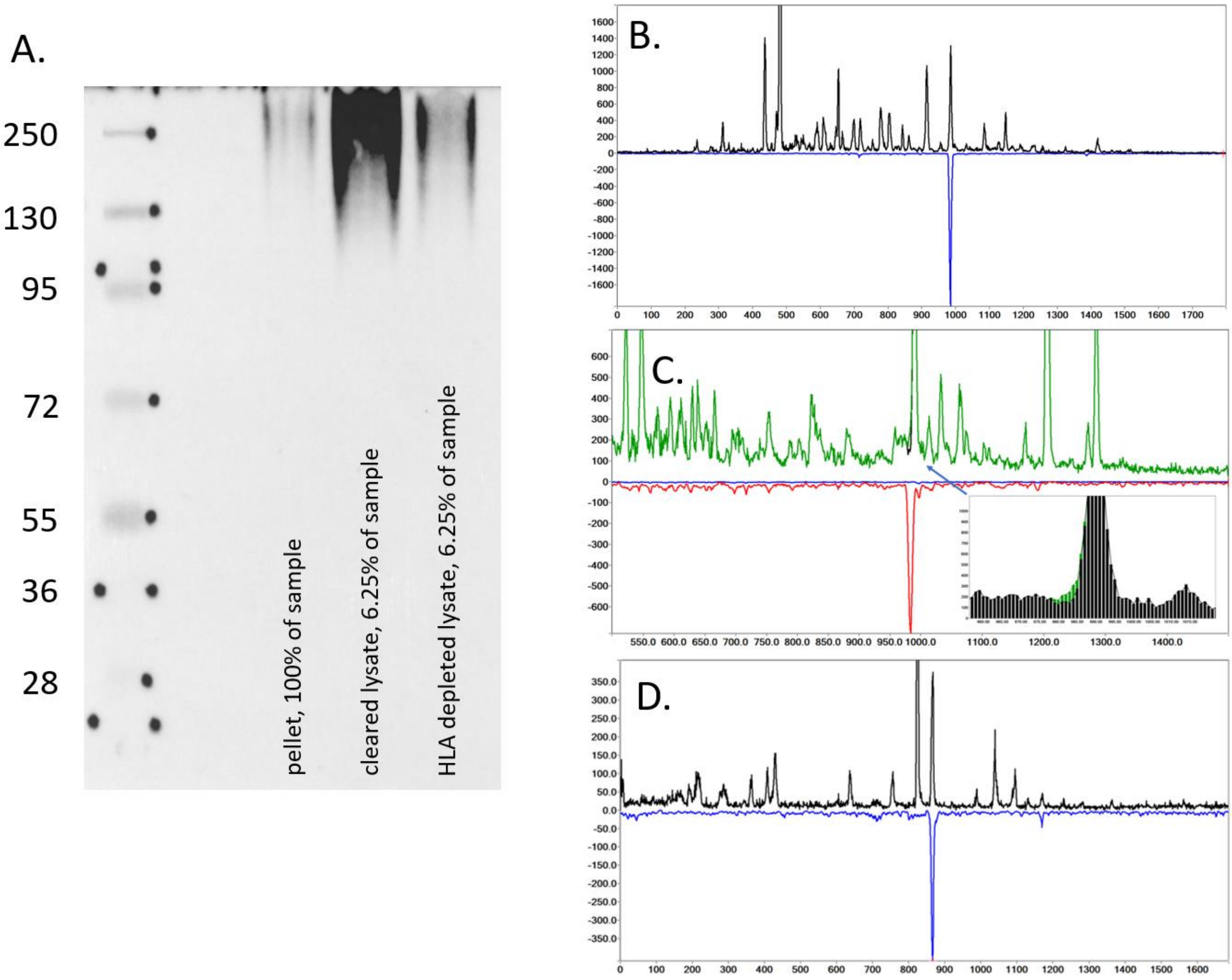

Continuation fig. 3

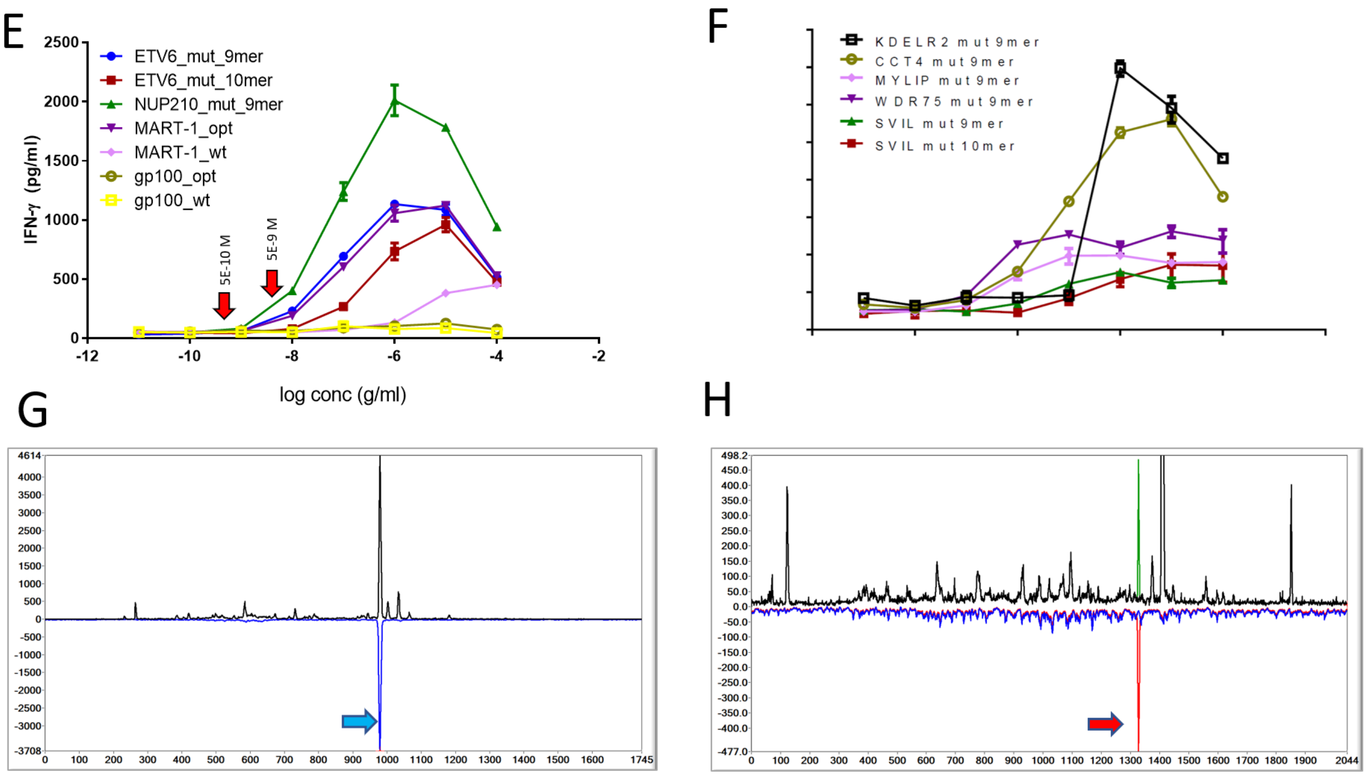

S3. *Determining the sensitivity of the MS method and the functional avidity of TIL*. Native gel western blot of cell lysate with pan HLA-A,B,C W6/32 Ab shows that peptide-HLA complexes are present in association with lipids and other proteins. The initial clearing pellet carries few pHLA complexes compared to the cleared lysate and there is almost complete removal of pHLA complexes from the lysate after affinity purification with W6/32 Ab immobilized on beads (A). Quantitation peptides are added to the acid which is then added to the beads for releasing peptides from the HLA. All downstream handling is shared between the endogenous and added peptides. A purified isotope labeled analogue of mutant MYLIP neoepitope was obtained and 100 attomoles added to the KADA immunoprecipitation beads at the acid elution step as an internal standard, and Poisson LC-DIAMS detection of the labeled MYLIP fragmentation pattern generates a dominating detection plot (B). This plot establishes elution position and ion precursor amplitude corresponding to 100 attomoles of the natural isotopic peptide if it were present. Scaled ion precursor and fragment events with amplitudes, elution profile and position defined by the isotope analogue but shifted masses are numerically inserted into the LC-DIAMS data by simulating a stochastic Poisson process to capture sampling (shot) noise (C). Black and blue traces are the Poisson detection plot for the data prior to insertion, while numerically adding 8 attomoles or 10 pHLA copies/cell of the natural isotope mutant MYLIP neoepitope is associated with a precursor amplitude of 124 counts/sec (overlaid green trace, differing only at the elution window) and a readily detectable Poisson signature (red) (C insert showing an expanded view around the elution profile). Poisson detection of 10 attomoles of MART-1 9mer AAGIGILTV added to beads containing affinity captured pHLA-A,B,C complexes from 425,000 ANRU cells (roughly 14 peptide

copies/cell) incubated with IFN-γ shows detection from the bead-immobilized pMHC complexes is sensitive (D).

All peptides recognized by TIL were titrated and added directly to TIL which was incubated 24 hours and thereafter secreted IFN-γ levels were analyzed, to establish the sensitivity of the responding KADA (E) or ANRU (F) TIL. The neoantigen inducing the best response in this assay was the mutant NUP210 peptide and since this peptide was not detected on ANRU by MS, this peptide could be loaded at 5 nM concentration (roughly 5x10-9 g/ml for 30 minutes). After the cells were washed and pHLA complexes affinity isolated, Poisson LC-DIAMS detection gave a dominating signature (G). Adding 50 attomoles of the NUP210 peptide to 1.25 ng of a commercial HeLa digest (as a carrier) and running this on our LC-DIAMS configuration gave a rough scale for the NUP210 abundance in the 5 nM loading experiment (375 attomoles recovered from 490,000 ANRU cells) as 460 copies/cell. Precursor and fragment ion signatures could be scaled down to 10% (5x10-10 g/ml) and numerically inserted into the LC-DIAMS dataset by a stochastic simulation of ion sampling over the predicted scans (by elution mapping, see Supplementary Figure 5) where mutant NUP210 elutes in a LCDIAMS run of the immune peptidome from 425,000 ANRU cells (H). The black trace is the extracted ion chromatogram for the NUP210 molecular ion and the blue trace is the Poisson amplitude for the NUP210 fragmentation pattern before numerical insertion of ion events and the green and red pair are the respective XIC and Poisson traces after insertion.

Supplementary fig 4

A.

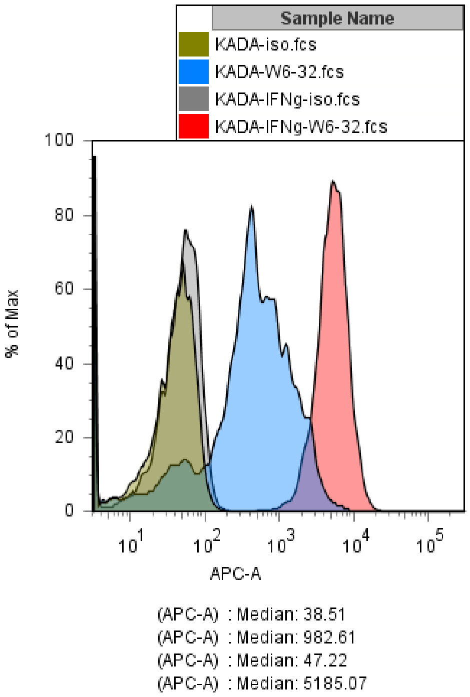

B.

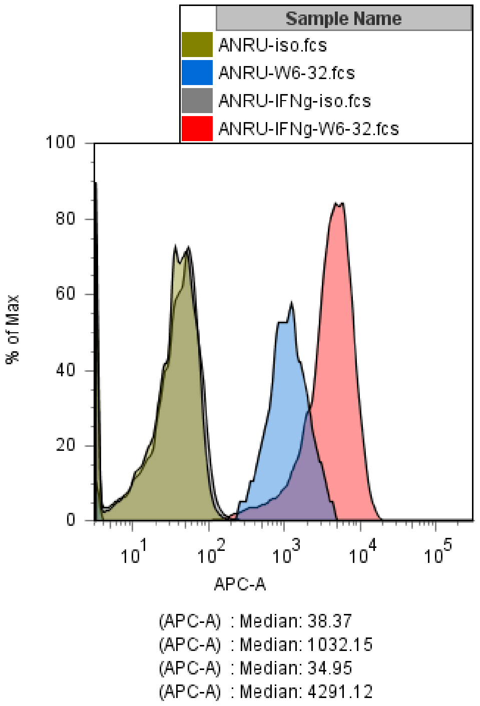

C.

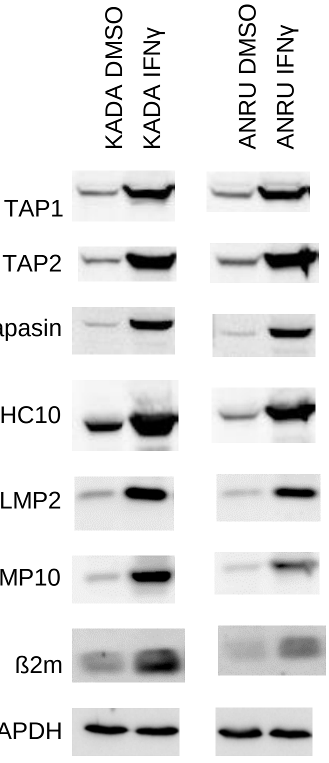

S4. *MHC class I upregulation by IFN-γ-treatment***.** KADA (A) or ANRU (B) tumor cells were cultured with or without addition of IFN-γ and MHC class I was analyzed by FACS. Western blot staining for peptide processing components in untreated and IFN-γ treated tumor cells (C).

Supplementary fig 5

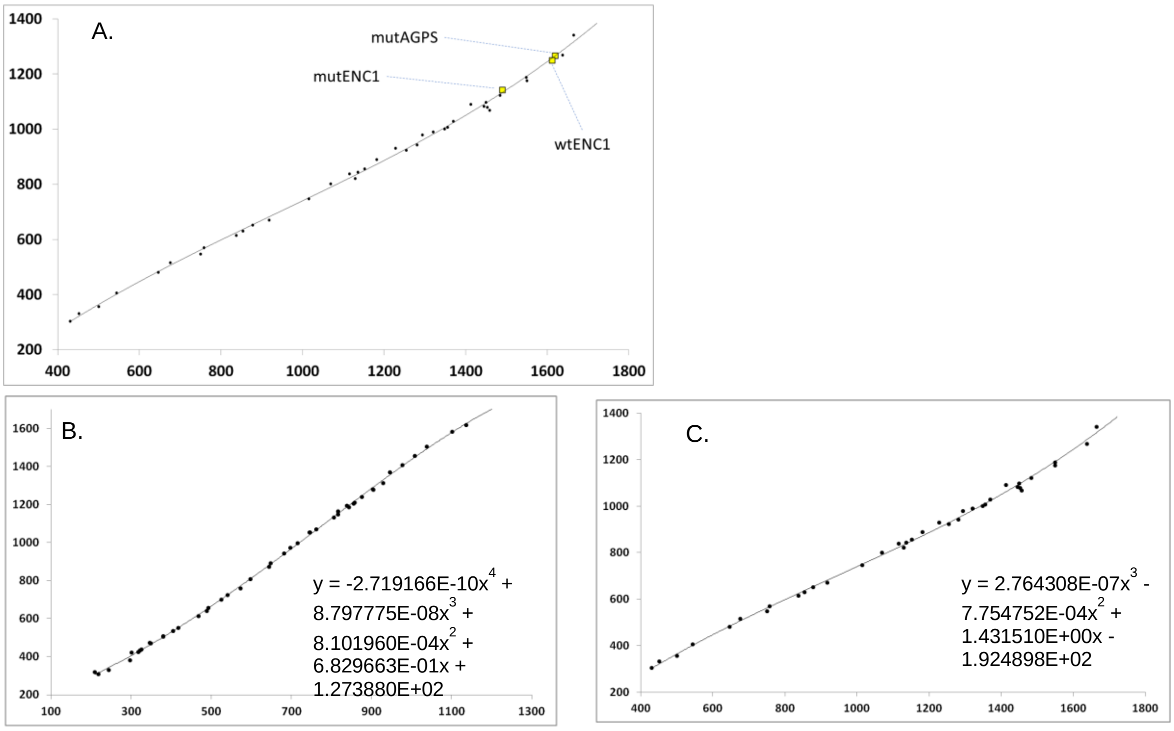

HeLa/synthetic -> HeLa/CaSki using tryptic peptides

HeLa/CaSki -> tumor sample using HLA-A02:01 peptides

S5. *Performing elution maps for detected peptides*. Having the set of synthetic neoantigens allows a precise prediction of their expected elution positions in the tumor sample. Reference fragmentation patterns for a set of easily available peptides (here HeLa peptides), HLA-A*02:01 immune peptides and a DIA run of HeLa peptides mixed with HLA-A*02:01 peptides are acquired once. The HLA-A*02:01$^+$ tumor sample is run first and then for each set of predicted neoantigens, a DDA run of the synthetic peptides identifies their fragmentation patterns. The synthetic peptides are then mixed 1.25 ng of the HeLa digest and the mixture is analyzed by Poisson LC-DIAMS for the elution positions of the synthetic peptides (set (10)) using the DDA reference patterns and the elution positions of the HeLa peptides (set (11)) using the HeLa reference patterns. The HeLa peptide positions in the HeLa + A2 DIA REF have been determined (set (10)) and the paired HeLa positions {H1, H2} define a mapping $f_1(x)$ from DIA RUN to DIA REF. The paired elution positions of the A2 peptides in DIA REF and the tumor sample {A1, A2} define a second mapping $f_2(x)$ that maps elution positions in DIA REF into elution positions in the tumor sample. Then the composition of maps $f_2(f_1((12)))$ identifies the elution of the synthetic peptides in the tumor sample. The maps, determined on a common LC configuration, show little scatter, and give a precise fix on the positions of the Poisson detection peaks.

Supplementary tables

Table S1. In-depth information about selection of mutated peptides predicted to bind HLA-A2 with high affinity from whole exome sequences of tumor cell lines.

		Patient	
		KADA	ANRU
		Number of mutations	
Mutation Type	*Total*	13127	1706
	In ORF	3958	481
SNV	*Nonsynonymous*	2554	323
	aa exchange	2407	308
	Stop codon gain	147	14
	Stop codon loss	0	1
DNV, TNV	*Total*	389 (4 TNVs)	30
	In ORF	120 (2 TNVs)	9
	Nonsynonymous	120	9
	aa exchange	112 (2 TNVs)	8
	Stop codon gain	8	1
InDels	*Total*	14644	14693
	In exons	571	611
	Non-frame-shift deltion	162	176
	Non-frame-shift insertion	123	136
	Frame-shift deletion	99	122
	Frame-shift insertion	103	95
	Stop codon gain	6	6
	Unknown	78	77

SNV, Single-nucleotide variant; DNV, Double-nucleotide variant; TNV, Triple-nucleotide variant; InDels, Insertion/Deletion; ORF, Open reading-frame.

Table S2. Expression of mutated alleles

mutation	position (hg19, genomic plus strand)	Sanger seq. – electropherogram detail
ANRU tumor cell line		
ETV6_L340V	chr12:12037387 C>G	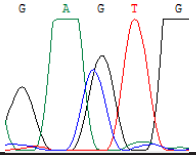
NUP210_S427F	chr3:13417804 G>A	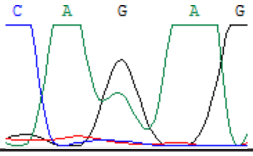
KADA cell line		
AGPS_P533L	chr2:178372750 C>T	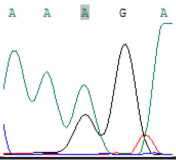
CCT4_S115F	chr2:62107456 C>T	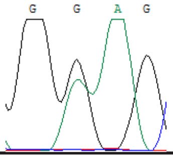
ENC1_P230S	chr5:73931623 C>T	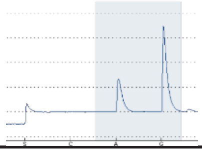
KDELR2_T121I	chr7:6505943-6505944 CC>TT	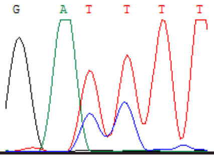
MYLIP_P8L	chr6:16129576 C>T	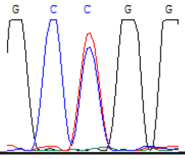
SVIL_E2183K	chr10:29747374 C>T	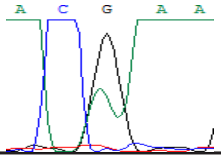
WDR75_S749F	chr2:190339492 C>T	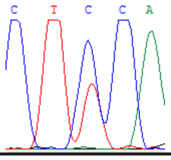

Table S2. Neoepitopes that elicited a T-cell response were selected to confirm the expression of the mutated allele by Sanger sequencing of the RT-PCR product. The 2nd column lists the genomic position (hg19 human reference genome version) and the base mutation, at the genomic plus strand, that was identified from the tumor cell line's exome. The 3rd column depicts the mutation as seen in Sanger sequencing data of the RT-PCR product of the respective mutation (the RT-PCR product was sequenced in both directions; depicted is a detail of the qualitatively superior electropherogram). The mutation in the gene ENC1 (KADA cell line) was validated by pyrosequencing instead of Sanger sequencing. The allele frequency of the mutated, ENC1_P230S-encoding allele was measured to be 51%. For all mutations validates by RT-PCR plus Sanger sequencing, both, wild type and mutated alleles are expressed.

Table S3. Functional avidity of T cell responses for mutated and wild type tumor- associated antigen-derived peptides

Patient	Peptide	logEC50	Top concentration
KADA	*KDELR2 9 mer*	-6.695	3002
	CCT4 9 mer	-6.415	1644
	MYLIP 9 mer	-8.187	119.2
	SVIL 9 mer	-7.489	83.3
	SVIL 10 mer	-6.226	106.9
	WDR75 9 mer	-8.496	176.4
	Influenza	-7.82	247.3
ANRU	*ETV6 9 mer*	-7.216	1136
	ETV6 10 mer	-6.454	987
	NUP210 9 mer	-7.294	1928
	MART-1 optimized	-7.033	1138
	MART-1 wt	-5.452	475

Mutation= the somatic mutation; the position is according to the main NCBI Genbank transcript. HLA-A*02 affinity (netMHCpan 2.8) is given as IC50 in nM (refs. 8 and 9).

Table S4. PCR primers used for Realtime RT-qPCR and analysis of mutated allele expression by Sanger Sequencing of RT-PCR products.

Patient	Forward primer	Reverse primer
Realtime RT-qPCR housekeeping gene controls:		
GAPDH	GAAGGTGAAGGTCGGAGTC	GAAGATGGTGATGGGATTTC
Actin	AGCCTCGCCTTTGCCGA	CTGGTGCCTGGGGCG
RPII	GCACCACGTCCAATGACAT	GTGCGGCTGCTTCCATAA
PPIA	CATCTGCACTGCCAAGACTGAG	TGCAATCCAGCTAGGCATG
Realtime RT-qPCR TAAs:		
MLANA/MART-1	TCATCGGGACAGCAAAGTGTC	GGTGAATAAGGTGGTGGTGACT
PMEL	GGCTGTGGTCCTTGCATCTC	CAGAAGATGCGGGGTAGACG
Realtime RT-qPCR mutated genes in ANRU tumor cell line:		
ETV6	CATGGTCTCTGTCTCCCCGC	GTTTTCGTACCGGCTGTCAGA
NUP210	GAGGGGACAGACGGCCATT	ATGTGAATTTCCACCTCCTGCT
Realtime RT-qPCR mutated genes in KADA tumor cell line:		
AGPS	GAGAATCTTTTGAGACTTCTGCTC	AGGAGCAAACTGAACACCCT
CCT4	GCAGGAGATGGCACCACAT	GGAATGACTCAGAAATGATGGTTGG
ENC1 (Realtime)	GACTTGGCCTCTCCGAAGTA	TCAGCACCTGGAAACATCTGC
ENC1 (allele analysis)	GTGCCAGCCTTACTGTCTGC	GGCTTGTGTACGAGTCTGCAA
KDELR2	AGTGGAGTTTCTGGTGGTCCC	GCGGAAGGATAGCCACGGAC
MYLIP (Realtime)	TGCTGCGAGGAGGAGATCAA	ATGCTCCACACGCGACC
MYLIP (allele analysis)	GGTCCCACCAGTGACAAGG	TCCGGTTTCTCAGGTTTAGCC
SVIL	GGGCGTTGTATTCATCCCTCGT	CCATTTACCCGCTGGCCGA
WDR75	ACCCTTAACAGAAAACATACCCGC	AGTCTCTTTAGACAGCAGCAATGA

SI References.

1. Li H & Durbin R (2009) Fast and accurate short read alignment with Burrows-Wheeler transform. *Bioinformatics* 25(14):1754-1760.
2. O'Rawe J*, et al.* (2013) Low concordance of multiple variant-calling pipelines: practical implications for exome and genome sequencing. *Genome Med* 5(3):28.
3. Cibulskis K*, et al.* (2013) Sensitive detection of somatic point mutations in impure and heterogeneous cancer samples. *Nat Biotechnol* 31(3):213-219.
4. Koboldt DC*, et al.* (2012) VarScan 2: somatic mutation and copy number alteration discovery in cancer by exome sequencing. *Genome Res* 22(3):568-576.
5. Wang K, Li M, & Hakonarson H (2010) ANNOVAR: functional annotation of genetic variants from high-throughput sequencing data. *Nucleic Acids Res* 38(16):e164.
6. Boegel S, Scholtalbers J, Lower M, Sahin U, & Castle JC (2015) In Silico HLA Typing Using Standard RNA-Seq Sequence Reads. *Methods Mol Biol* 1310:247-258.
7. Boegel S*, et al.* (2012) HLA typing from RNA-Seq sequence reads. *Genome Med* 4(12):102.
8. Lundegaard C*, et al.* (2008) NetMHC-3.0: accurate web accessible predictions of human, mouse and monkey MHC class I affinities for peptides of length 8-11. *Nucleic Acids Res* 36(Web Server issue):W509-512.
9. Nielsen M*, et al.* (2003) Reliable prediction of T-cell epitopes using neural networks with novel sequence representations. *Protein Sci* 12(5):1007-1017.
10. Aichinger G*, et al.* (2015) Phase I/II randomized double-blind study of the safety and immunogenicity of a nonadjuvanted vero cell culture-derived whole-virus H9N2 influenza vaccine in healthy adults. *Clin Vaccine Immunol* 22(1):46-55.
11. Keskin DB*, et al.* (2015) Physical detection of influenza A epitopes identifies a stealth subset on human lung epithelium evading natural CD8 immunity. *Proc Natl Acad Sci U S A* 112(7):2151-2156.
12. Respa A*, et al.* (2011) Association of IFN-gamma signal transduction defects with impaired HLA class I antigen processing in melanoma cell lines. *Clin Cancer Res* 17(9):2668-2678.
